# Effect of HA330 resin-directed hemoadsorption on a porcine acute respiratory distress syndrome model

**DOI:** 10.1186/s13613-017-0287-0

**Published:** 2017-08-14

**Authors:** Xuefeng Xu, Chune Jia, Sa Luo, Yanming Li, Fei Xiao, Huaping Dai, Chen Wang

**Affiliations:** 1 National Clinical Research Centre for Respiratory Diseases, Beijing Hospital, Beijing, 100730 China; 2 Department of Surgical Intensive Care Medicine, Beijing An Zhen Hospital, Capital Medical University, No. 2 Anzhen Rd., Chao-Yang District, Beijing, 100029 China; 3 Department of Pulmonary and Critical Care Medicine, China-Japan Friendship Hospital, 2 Yinghua Dongjie, Beijing, 100029 China; 4 Department of Pulmonary and Critical Care Medicine, Beijing Hospital, Beijing, 100730 China; 5 Key Laboratory of Geriatrics, Beijing Hospital and Beijing Institute of Geriatrics, Ministry of Health, Beijing, China

**Keywords:** ARDS, Cytokine storm, Hemoadsorption, Porcine model, Proteomics

## Abstract

**Background:**

Blood purification is an emerging approach to dampening the cytokine storm. This study aims to assess the efficacy of HA330 resin-directed hemoadsorption (HA) on endotoxin-induced porcine acute respiratory distress syndrome (ARDS) model.

**Methods:**

Twenty-four Chinese domestic pigs were allocated into saline group receiving intravenous infusion of saline (*N* = 6) and endotoxin group receiving intravenous infusion of LPS (*N* = 18). When ALI model was initially diagnosed, six pigs in the LPS and saline group were killed for BALF and histopathological analysis. The remaining 12 pigs in LPS group received 3-h HA (*N* = 6) or HA-sham (*N* = 6) treatment, respectively. Following another 5-h observation, animals were killed. Variables on hemodynamics, blood gases and lung mechanics were recorded at a series of time points. Differentially expressed cytokines and proteins were determined by ELISA and proteomics.

**Results:**

HA treatment significantly improved injured oxygenation induced by LPS. HA also partially improved the barrier permeability and reduced lung edema and inflammation/injury induced by LPS infusion. Proteomic analysis showed the differentially expressed proteins between HA- and HA-sham-treated groups mostly belonged to the categories of acute inflammation/immune response, and proteolysis.

**Conclusions:**

Hemoadsorption improved ARDS possibly by blunting the cytokine storm and by restoring homeostasis of the disordered proteome milieu in the exudative phase.

**Electronic supplementary material:**

The online version of this article (doi:10.1186/s13613-017-0287-0) contains supplementary material, which is available to authorized users.

## Background

The acute respiratory distress syndrome (ARDS) is a condition with the acute onset of noncardiac respiratory failure that develops in response to a series of insults to the alveolar-capillary barrier [1]. The current mainstay therapy for ARDS is largely supportive [2, 3]. This has been shown to reduce mortality by limiting further iatrogenic injury to the already injured lungs. However, the mortality rate of ARDS is still unacceptably high [4]. ARDS is largely caused by the local alveolar and circulating “cytokine storm” that happens with bacterial/virus infection, burn/trauma and some possible iatrogenic factors, such as high-volume ventilation [5]. This has encouraged the development of several specific targeted pharmacologic therapy directed against single key pathogenic mediators. However, most clinical trials have proven no benefit to disease outcome and were stopped early for futility [6]. Investigators shifted their interests into the extracorporeal blood purification (EBP) modality, which is a nonspecific, broad-spectrum method to blunt the “cytokine storm” by immediately clearing a series of circulating and resident mediators [7].

Until now, there are only some inconsistent preliminary data to investigate the effects of hemofiltration (a modality of EBP) on ARDS animal models [8, 9]. Also, it has been shown that hemoadsorption (HA), another modality of EBP, can improve oxygenation in septic patients [10, 11]. However, evidences of HA’s effects on ARDS patients animal models are scarce. We hypothesize that HA can improve oxygenation, reduce the permeability of alveolar-capillary barrier and alleviate pulmonary edema/damage by blunting the circulating/alveolar cytokines in the process of ARDS. To test this hypothesis, we established an endotoxin-induced ARDS porcine model to explore HA’s therapeutic effect by using a HA330 resin cartridge (a new weapon against cytokine storm).

## Methods

### Research animals and ethical considerations

This study was approved by the Institutional Animal Experiment Committee of Beijing Hospital. All experiments were performed according to the Declaration of Helsinki conventions for the use and care of animals. Twenty-four healthy, 3–4-week-old Chinese domestic white pigs of both sexes were chosen from a local stock routinely used for the experimental research. The average weight of pigs used in this experimental procedure is 43 ± 0.3 kg (mean ± SEM).

### Animal preparations

#### Sedation, anesthesia and muscle relaxation

Detailed information is shown in Additional file 1: Supplement-Methods.

#### Ventilation and measurements of lung mechanics

Animals were ventilated in a volume-controlled mode. The ventilation protocol at baseline used a tidal volume of 10 ml/kg body weight, a fraction of inspired oxygen (FiO_2_) of 0.4 and a positive end-expiratory pressure (PEEP) at 0 cmH_2_O. Parameters of lung mechanics were recorded. Detailed information is shown in Additional file 1: Supplement-Methods.

#### Instrumentation and hemodynamic measurements

Cardiac output (CO), mean arterial pressure (MAP), systematic vascular resistance (SVR), mean pulmonary arterial pressure (MPAP), pulmonary vascular resistance (PVR), pulmonary artery wedge pressure (PAWP), extravascular lung water (EVLW) and pulmonary vascular permeability index (PVPI) were measured with PiCCO and Swan-Ganz systems. Detailed information is shown in Additional file 1: Supplement-Methods.

### Experimental protocol

After surgical preparations and instrumentation, all animals were ventilated in prone position and a period of 1 h was left for animal stabilization. Thereafter, baseline (i.e., the time point after 1-h stabilization) parameters including lung mechanics, hemodynamics, blood gases were recorded and venous blood samples were collected. As a next step, lung injury model was produced with i.v. infusion of 50 μg/kg LPS (*Escherichia coli*, serotype O55:B5, Sigma-Aldrich, St. Louis, MO, USA) mixed in 500 ml of saline that was delivered via a volumetric infusion pump (Instrumentation Laboratory, Bedford, MA, USA) for about 2 h [12, 13]. Before the start of the experiment, 24 animals were purchased and randomly divided into four groups according to different conditions: (a) control group (*N* = 6), animals received an i.v. infusion of saline without LPS (LPS-sham); (b) LPS group (*N* = 6), animals received an i.v. infusion of saline with LPS as indicated above. When experimental ARDS was diagnosed in LPS group (PaO_2_/FiO_2_ is equal or less than 200 mmHg with a PEEP equal to 5 cmH_2_O), animals in control and LPS group were all killed for bronchoalveolar lavage fluid (BALF) and histologic assessment in order to aid further diagnosis of lung injury. (c) LPS plus HA-sham group (*N* = 6), animals received an i.v. infusion of saline with LPS. Immediately after ARDS was well established, HA-sham treatment was started by using a hollow fiber membrane hemofilter (Fresenius) with the ultrafiltration line clamped. Two animals in this group were excluded because they died or developed severe sepsis/septic shock and refractory acidosis despite active liquid recovery and vasopressor administration before moderate ARDS diagnosis; (d) LPS plus HA group (*N* = 6), animals received an i.v. infusion of saline with LPS, and real HA treatment was started by using a disposable hemoperfusion cartridge (HA330 resin, styrene divinylbenzene copolymers, with a blood flow of 200–250 ml/min, volume of 185 ml; Jafron Biomedical Co., Ltd., Zhuhai, GuangDong, China; http://www.jafron.com/). Two pigs were also discarded because they died of septic shock and severe disturbance of water, electrolyte and acid base before the establishment of hemoperfusion circulation. HA-sham and HA treatment lasted for 3 h, and thereafter, the animals were monitored for an extended observation period of 5 h and then killed with a bolus injection of 10 ml of 15% KCl. BAL fluid collection and lung tissue harvesting were performed immediately upon exsanguination and sternotomy performance for the measurements of inflammatory markers, proteomes and for histologic evaluation. Parameters of hemodynamics, blood gases and lung mechanics were recorded every hour following the start of LPS or sham LPS infusion until the end of experiment. Plasma samples were also prepared in the corresponding time points. During the experiment, all pigs were continuously infused with Ringer lactate solution or saline at a rate of 12 ml/kg/h at the start of the experiment. The infusion rate was increased to 15–20 ml/kg/h if the MAP was less than 70–50 mmHg. However, if pulmonary artery wedge pressure (PAWP) continuously raised and exceeded 12 mmHg, the rate of infusion should be reduced to prevent fluid overload. Meanwhile, norepinephrine (0.5–1.5 μg/kg/min) was then used to achieve this level of mPAP in LPS-treated pigs. Body temperature was maintained over 36 °C by using a heating blanket. The experimental protocol was summarized in Additional file 2: Fig. S1.

### Assessment of the LPS-induced ARDS porcine model

To determine whether ARDS has occurred, at least four main features of ARDS should be assessed: (1) abnormalities of gas exchange. A ratio of the partial pressure of arterial oxygen to the fraction of inspired oxygen (PaO_2_/FiO_2_) ≤ 200 mmHg at PEEP = 5 cmH_2_O (clinical diagnostic criteria of moderate ARDS) is the initial evaluating criteria;(2)a remarkable leakage of alveolar barrier as indicated by the significant increase of lung wet-to-dry weight ratio, EWLW and PVPI; (3) a significant infiltration of inflammatory cells and production of inflammatory cytokines in both circulation and lung tissues (plasma and lung IL-6, IL-8, IL-1β, IL-17A and TNF-α levels were measured in our study); (4) diffuse alveolar damage (DAD) as determined by lung histopathological examination [14].

### HA330 cartridge-directed hemoadsorption

The HA330 is an electrically neutral microporous resin that is a powerful new weapon in the clearance of “cytokine storm” occurred in sepsis [10]. Detailed information is shown in Additional file 1: Supplement-Methods.

### BALF recovery

Sixty milliliter ice-cold saline was used to collect BALF in the left upper, middle and lower lobes. Detailed information is shown in Additional file 1: Supplement-Methods.

### Assessment of total protein contents in BALF

BCA (bicinchoninic acid) protein assay reagent kit (Pierce Biotechnology, Rockford, IL, USA) was used. Detailed information is shown in Additional file 1: Supplement-Methods.

### Lung tissue collection and histopathological evaluation

The right lower lobe was excised, fixed and sliced for routine hematoxylin and eosin (H&E) staining. ALI scoring was done according to a consensus report published previously [14] (Additional file 3: Table S1). Detailed information is shown in Additional file 1: Supplement-Methods.

### Lung water assessed by gravimetric method

Samples from unused right lung lobes were dissected free of non-lung parenchymal tissue, placed in a dish and weighed (wet weight). Then, they were dried in an oven at 80 °C and weighed daily until their weight was maintained unchanged (dry weight). The total water content of the lung was crudely estimated as a wet-to-dry weight ratio (W/D).

### Concentrations of proinflammatory mediators in circulation and lung tissues

IL-1β, IL-6, IL-8, TNF-α and IL-17A were measured by ELISA kits (Bluegene Biotech CO., LTD, Shanghai, China). Detailed information is shown in Additional file 1: Supplement-Methods.

### Plasma and lung proteome

Protein samples of plasma, BALF and lung tissue in HA or HA-sham treatment group were collected and received iTRAQ-labeled (Applied Biosystems, Foster City, CA, USA) mass spectrometric analysis. Bioinformatics analysis was also performed [15, 16]. Detailed information is shown in Additional file 1: Supplement-Methods.

### Statistical analysis

The data are shown as the mean ± SEM. Parametric data between multiple groups were compared by using the Kruskal–Wallis test and followed by Mann–Whitney *U* test if statistically significant. Mann–Whitney *U* test was also used for the comparison between two independent continuous variables. Data were analyzed using the SPSS statistical software package for Windows, version 13.0 (SPSS, Chicago, IL, USA), and *P* < 0.05 was considered as statistically significant.

## Results

### Intravenous infusion of endotoxin causes sharp changes in physiological parameters

**Fig. 1:**
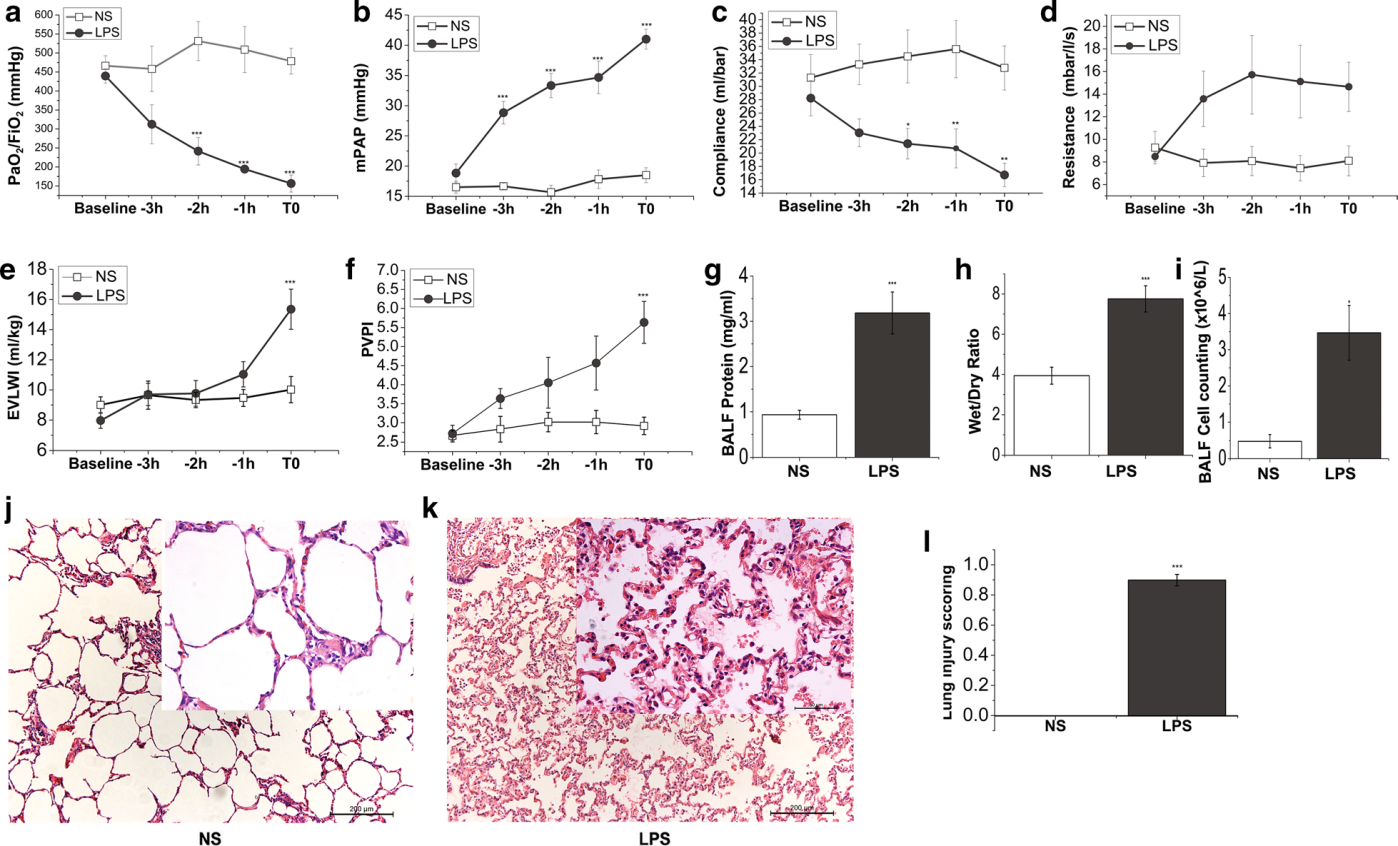
LPS impairs lung function and alveolar-capillary barriers. Pigs were allocated into intravenous saline and LPS group, respectively. Parameters of oxygenation, hemodynamics and lung mechanics were recorded at different time points from baseline to the end of the experiment (T0). BALF and lung tissues were collected at T0. PaO_2_/FiO_2_ (**a**) was measured by using blood samples from femoral artery on a blood gas analysis instrument. The value of mPAP (**b**) was gained by Swan-Ganz catheter implantation into jugular vein. Compliance (**c**) and resistance (**d**) were recorded by using Drager Evita 4 ventilator. EVLWI (**e**) and PVPI (**f**) were measured by PiCCO system at baseline and a series of time points until ALI was well established (T0). **g** Whole protein content in the BALF of pigs was detected by using BCA protein assay kit. **h** The ratio of wet/dry lung weight of saline control and LPS challenge pigs. **i** Whole-cell counts in the BALF collected from both groups of pigs. **j** and **k** were the representative histology sections of controls and LPS challenge pigs. HE sections are from one representative animal per group. **l** Lung injury scores were calculated according to ATS report which was in detail described in the “Methods.” Data points are expressed as mean ± SEM, *N* = 6 of each group. Statistical significance between saline and LPS group per point of measurement is shown as *P < 0.05; **P < 0.01; ***P < 0.001, respectively

The ratio of PaO_2_/FiO_2_ and alveolar-arterial oxygen partial pressure difference (AaDO_2_) in pigs that received LPS infusion declined gradually (from 440 ± 19 to 157 ± 23 mmHg, *P* < 0.001, Fig. 1a; Table 1). After LPS infusion, CO was firstly increased and then decreased when moderate ARDS was diagnosed. However, the changes did not reach statistical difference when compared with saline group. LPS infusion increased MPAP (18.8 ± 1.5 vs. 41.0 ± 1.7 mmHg, *P* < 0.001, Fig. 1b; Table 2) and PVRI (201 ± 13 vs. 781 ± 74 dyn s/cm^5^/m^2^, *P* < 0.001, Table 2). However, MAP and SVRI continued to decrease (Table 2). In addition, pulmonary mechanics were also damaged as shown by an increase in P_AW_Peak, P_AW_Plateau and resistance and a decrease in pulmonary static compliance (Fig. 1c, d; Table 1).

**Table 1:** Variables determining oxygenation and lung mechanics in saline controls and LPS-challenged animals

Parameter	Group	Time (h)				
		Baseline	−3 h	−2 h	−1 h	T0
PaO_2_/FiO_2_ (mmHg)	NS	466 ± 26	458 ± 60	531 ± 51	509 ± 60	479 ± 34
	LPS	440 ± 19	312 ± 51	242 ± 36***	194 ± 10***	157 ± 23***
PaCO_2_ (mmHg)	NS	35.2 ± 2.8	30.8 ± 2.8	30.5 ± 1.8	32.3 ± 1.1	31.8 ± 1.49
	LPS	36.8 ± 2.4	30.2 ± 1.8	37.5 ± 1.5	36.5 ± 3.0	44.7 ± 5.0**
AaDO_2_ (mmHg)	NS	47 ± 7	48 ± 14	52 ± 1	51 ± 12	60 ± 4
	LPS	63 ± 5	124 ± 20	143 ± 15**	213 ± 53**	224 ± 48**
P_AW_Peak (mbar)	NS	16.5 ± 1.8	16.5 ± 1.5	16.5 ± 1.7	17.3 ± 2.0	17.3 ± 1.8
	LPS	21.3 ± 0.9	27.2 ± 1.8**	30.2 ± 2.4***	31.2 ± 2.4***	35.7 ± 2.5***
P_AW_Plateau (mbar)	NS	12.2 ± 1.8	11.5 ± 1.7	12.2 ± 2.0	13.2 ± 2.3	13.8 ± 2.2
	LPS	15.3 ± 1.2	18.8 ± 1.4	22.3 ± 2.3**	25.7 ± 2.5***	27.3 ± 3.2***
Resistance (mbar/l/s)	NS	9.3 ± 1.4	7.9 ± 1.2	8.1 ± 1.3	7.5 ± 1.1	8.1 ± 1.3
	LPS	8.5 ± 0.4	13.6 ± 2.5	15.7 ± 3.5	15.1 ± 3.2	14.7 ± 2.2
Compliance (ml/mbar)	NS	31.2 ± 3.5	33.3 ± 3.0	34.5 ± 4.0	35.6 ± 4.3	32.8 ± 3.3
	LPS	28.2 ± 2.7	23.0 ± 2.1	21.4 ± 2.3*	20.7 ± 2.9**	16.7 ± 1.8**

Oxygenation and ventilation parameters in saline control and LPS challenge animals at baseline and a series of time points until the end of the experiment when ALI model was diagnosed (data shown are mean ± SEM, *N* = 6 of each group; *P < 0.05; **P < 0.01; ***P < 0.001 versus control group). PaO_2_, oxygen partial pressure; FiO_2_, inspiratory oxygen fraction; PaCO_2_, carbon dioxide partial pressure; P_AW_Peak, peak airway pressure; P_AW_Plateau, plateau airway pressure

**Table 2:** Systemic and pulmonary hemodynamic measurements in saline controls and LPS-challenged animals

Parameter	Group	Time (h)				
		Baseline	−3 h	−2 h	−1 h	T0
CO (l/min)	NS	4.1 ± 0.3	4.5 ± 0.4	4.1 ± 0.4	4.2 ± 0.4	4.2 ± 0.4
	LPS	4.1 ± 0.3	4.1 ± 0.5	5.1 ± 0.3	5.0 ± 0.3	3.4 ± 0.3
MAP (mmHg)	NS	105 ± 6	98 ± 6	102 ± 8	101 ± 7	102 ± 8
	LPS	113 ± 3	78 ± 8	56 ± 4***	57 ± 5***	54 ± 5***
SVRI (dyn s/cm^5^/m^2^)	NS	1783 ± 198	1810 ± 169	1927 ± 148	1822 ± 183	1909 ± 218
	LPS	2399 ± 238	1987 ± 217	1002 ± 111**	952 ± 56**	1160 ± 97*
mPAP (mmHg)	NS	16.5 ± 1.0	16.7 ± 0.5	15.7 ± 1.2	17.8 ± 1.5	18.5 ± 1.2
	LPS	18.8 ± 1.5	28.8 ± 1.9***	3.3 ± 2.0***	34.7 ± 2.7***	41.0 ± 1.7***
PVRI (dyn s/cm^5^/m^2^)	NS	204 ± 20	193 ± 16	231 ± 48	209 ± 35	201 ± 13
	LPS	364 ± 40	552 ± 115***	542 ± 67**	634 ± 120***	781 ± 74***
PAWP (mmHg)	NS	6.0 ± 1.2	6.5 ± 1.5	6.3 ± 1.4	6.8 ± 1.5	6.8 ± 1.2
	LPS	4.0 ± 1.2	5.5 ± 0.6	8.0 ± 0.6	9.0 ± 0.9	12.2 ± 1.4*
EVLWI (ml/kg)	NS	9.0 ± 0.5	9.7 ± 0.9	9.3 ± 0.5	9.5 ± 0.6	10.0 ± 0.9
	LPS	8.0 ± 0.5	9.7 ± 0.7	9.8 ± 0.9	11.0 ± 0.8	15.4 ± 1.3***
PVPI	NS	2.7 ± 0.1	2.8 ± 0.3	3.0 ± 0.3	3.0 ± 0.3	2.9 ± 0.2
	LPS	2.7 ± 0.2	3.6 ± 0.3	4.0 ± 0.7	4.6 ± 0.7	5.6 ± 0.5***

Hemodynamic parameters are compared between the saline control and LPS-challenged group. (Data are mean ± SEM, *N* = 6 of each group; *P < 0.05, **P < 0.01, ***P < 0.001; versus control group)

*CO* cardiac output, *MAP* mean arterial pressure, *SVRI* systemic vascular resistance index, *MPAP* mean pulmonary arterial pressure, *PVRI* pulmonary vascular resistance index, *PAWP* pulmonary artery wedge pressure, *EVLWI* extravascular lung water index, *PVPI* pulmonary vascular permeability index

### Intravenous infusion of endotoxin causes high-permeability pulmonary edema and lung histologic damages

The EVLWI continuously increased after LPS infusion until ARDS was diagnosed (from 8.0 ± 0.5 ml/kg in baseline to 15.4 ± 1.3 ml/kg at the end of this experiment when ARDS was established, *P* < 0.001; Fig. 1e). In parallel, there was also a remarkable increase in lung wet/dry ratio in LPS-challenged pigs compared with saline group (Fig. 1h). The alveolar-capillary membrane was also seriously injured by LPS infusion as manifested by a significant increase in both pulmonary vascular permeability index (PVPI) and whole BAL protein concentration (Fig. 1f, g). Infusion of LPS in the endotoxin challenge group was also associated with an increase in the numbers of whole BAL cell counting (Fig. 1i). To determine the histopathological features of ALI model, subset of animals (*N* = 6 in LPS and saline group, respectively) were killed when ARDS model was diagnosed based on oxygenation parameter (PaO_2_/FiO_2_ ≤ 200 mmHg at PEEP = 5 cmH_2_O). Histologic examinations from animals receiving LPS demonstrated alveolar bleeding, microatelectasis, perivascular edema, marked leukocyte sequestration in alveolar septa and lumen, thickened alveolar walls, and the presence of proteinaceous debris in the alveolar space as compared to control subjects (Fig. 1j–l, Additional file 4: Fig. S2).

### Intravenous infusion of endotoxin augments the expressions of systemic and pulmonary inflammatory cytokines

**Fig. 2:**
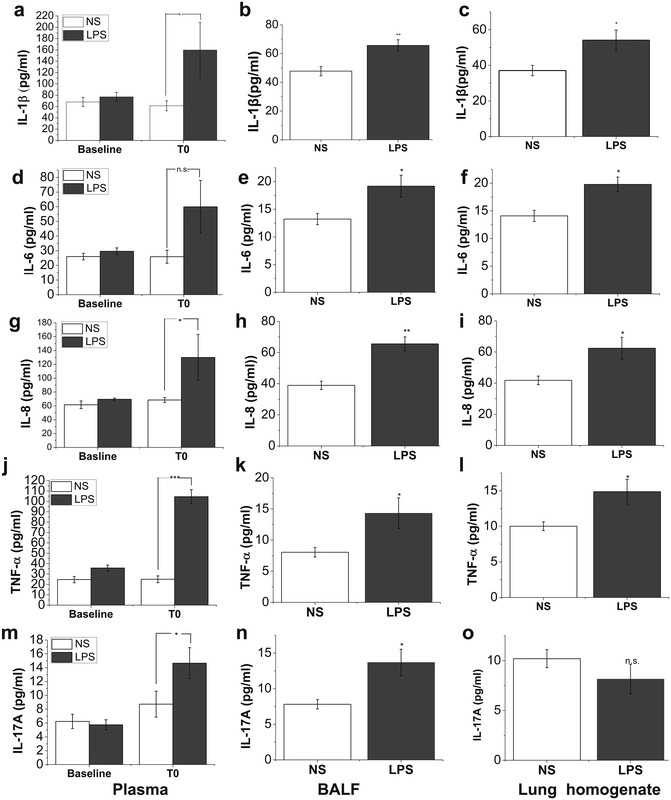
Concentrations of circulating and pulmonary inflammatory parameters. In LPS group, plasma samples were collected before LPS challenge (baseline) and at the time when ARDS model had been well established (T0). BALF and lung homogenates were also prepared at necropsy. Samples from saline control group were also obtained at the corresponding time points. The level of circulating and pulmonary IL-1β (**a**–**c**), IL-6 (**d**–**f**), IL-8 (**g**–**i**), TNF-α (**j**–**l**) and IL-17A (**m**–**o**) was detected by using commercial ELISA kits. Data are presented as mean ± SEM (*N* = 6 of each group). Compared with saline (NS) group, *P < 0.05; **P < 0.01; ***P < 0.001

As shown by ELSA assay in Fig. 2, there was no difference in the baseline concentration of plasma IL-1β (Fig. 2a), IL-6 (Fig. 2d), IL-8 (Fig. 2g), TNF-α (Fig. 2j) and IL-17A (Fig. 2m) between LPS and saline groups, but all of these cytokines increased rapidly when challenged with LPS infusion at T0 compared with saline infusion. We also determined the levels of these inflammatory cytokines within BALF and lung homogenates to assess the inflammatory response in the alveolar compartment. ELISA assay showed significant increased levels of these cytokines in BALF and lung homogenates after LPS challenge except IL-17A, which was remarkable increased in BALF but remained unchanged in lung homogenates at T0 (Fig. 2).

### Hemoadsorption improves oxygenation and lung mechanics in pigs challenged with endotoxin

**Fig. 3:**
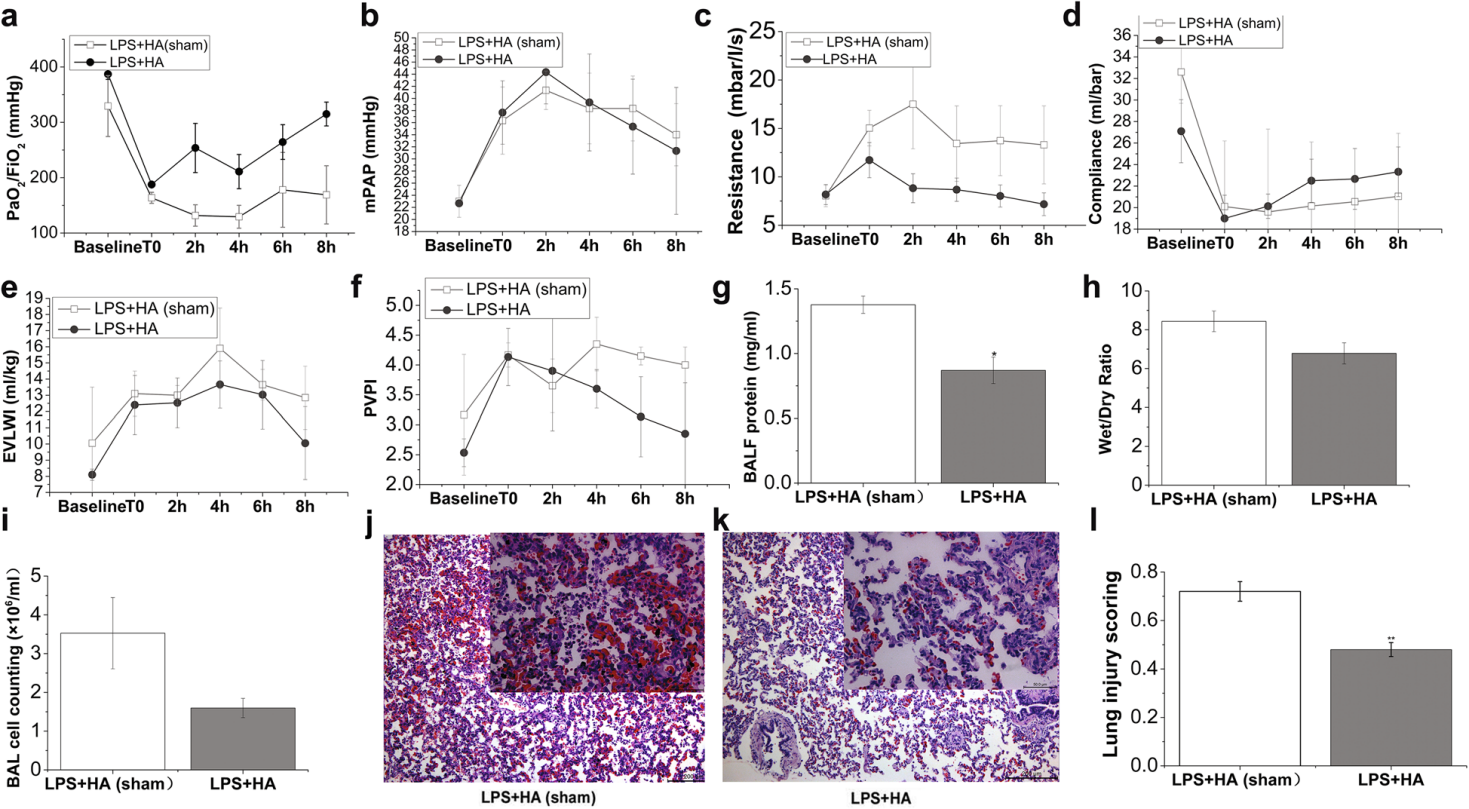
Effects of HA on oxygenation, lung mechanics and alveolar-capillary barriers. Pigs were challenged with LPS (50 μg/kg) intravenously to recapitulate the features of ARDS. When ARDS was well established (T0), animals received hemoadsorption (HA) or sham-hemoadsorption treatment for 3 h. Following another 5-h observation, animals were killed for BALF and lung tissue processing. At baseline, T0, and a series of time points during (2 h) and after HA (or HA-sham) treatment (4, 6 and 8 h), parameters of oxygenation and lung mechanics were recorded. PaO_2_/FiO_2_ (**a**) was measured by using blood samples from femoral artery on a blood gas analysis instrument. The value of mPAP (**b**) was gained by Swan-Ganz catheter implantation into jugular vein. Resistance (**c**) and compliance (**d**) were recorded by using Drager Evita 4 ventilator. EVLWI (**e**) and PVPI (**f**) were measured by PiCCO system at indicated time points (baseline, T0, 2, 4, 6 and 8 h following HA or HA-sham treatment). BALF protein (**g**) was assessed at 8 h after HA (or HA-sham) as described in “Methods”. **h** The ratio of wet/dry lung weight of animals in both groups was calculated by gravimetric method. **i** Whole-cell counts in the BALF collected from both groups of pigs were numerated by using a counting plate. **j** and **k** were the representative lung histology sections of pigs in LPS + HA (sham) and LPS + HA groups. HE sections are from one representative animal per group. **l** Lung injury scores were calculated according to ATS report which was in detail described in the “Methods.” Data points represent as mean ± SEM, *N* = 3–4 of each group and time point. Statistical significance between HA and HA-sham group per point of measurement is shown as *P < 0.05

We found that treatment with HA increased LPS-impaired oxygenation (387 ± 10 mmHg at baseline vs. 183 ± 6 mmHg at T0 vs 315 ± 22 mmHg at 8 h after HA; Fig. 3a; Table 3). In contrast, in endotoxin-challenged pigs that did not receive HA (HA-sham) treatment, the ratio of PaO_2_/FiO_2_ was only slightly increased at 8 h after HA-sham (Fig. 3a; Table 3). Treatment with HA significantly or showed a trend toward decreasing P_AW_Peak, P_AW_Plateau, lung resistance and the impairment of static compliance induced by endotoxin infusion when all pigs data were pooled together and compared with HA-sham group (Fig. 3c, d; Table 3). However, HA did not alter the impairment of systemic/pulmonary hemodynamic parameters (Fig. 3b; Table 4).

**Table 3:** Variables determining oxygenation and lung mechanics in LPS + HA (sham)- and LPS + HA-treated animals

Parameter	Group	Time (h)					
		Baseline	T0	2 h	4 h	6 h	8 h
PaO_2_/FiO_2_ (mmHg)	LPS + HA (sham)	329 ± 55	164 ± 10^†^	131 ± 20^†^	129 ± 21^†^	178 ± 68	169 ± 53
	LPS + HA	387 ± 10	183 ± 6^†^	254 ± 44*	211 ± 31^†^	264 ± 31	315 ± 22*^§^
PaCO_2_ (mmHg)	LPS + HA (sham)	45 ± 4	40 ± 7	45 ± 17	46 ± 14	46 ± 16	42 ± 14
	LPS + HA	37 ± 5	53 ± 6^†^	59 ± 9^†^	54 ± 3^†^	51 ± 2	45 ± 3
AaDO_2_ (mmHg)	LPS + HA (sham)	96 ± 24	170 ± 6	363 ± 129	330 ± 103	371 ± 102^†^	358 ± 96
	LPS + HA	79 ± 10	172 ± 29^†^	368 ± 68^†^	368 ± 77^†^	334 ± 81^†^	296 ± 66^†^
P_AW_Peak (mbar)	LPS + HA (sham)	17.7 ± 1.8	32.0 ± 2.6^†^	29.7 ± 5.2^†^	29.7 ± 2.7^†^	28.3 ± 1.7^†^	26.7 ± 3.2
	LPS + HA	18.9 ± 1.5	29.0 ± 1.0^†^	26.3 ± 1.9^†^	26.3 ± 1.8^†^	24.7 ± 1.8	24.3 ± 2.3
P_AW_Plateau (mbar)	LPS + HA (sham)	13.3 ± 0.9	21.0 ± 1.0^†^	22.7 ± 2.9^†^	23.3 ± 2.4^†^	21.0 ± 1.0^†^	21.7 ± 0.9^†^
	LPS + HA	15.3 ± 1.8	24.7 ± 1.3^†^	22.0 ± 1.2^†^	21.0 ± 1.0^†§^	20.3 ± 0.9^†§^	19.0 ± 0.0*^†§^
Resistance (mbar/l/s)	LPS + HA (sham)	8.0 ± 1.1	15.0 ± 1.8	17.5 ± 4.6	13.4 ± 3.9	13.7 ± 3.6	13.3 ± 4.0
	LPS + HA	8.2 ± 1.0	11.7 ± 1.8	8.8 ± 1.5	8.7 ± 1.2	8.0 ± 1.1	7.2 ± 1.2^§^
Compliance (ml/mbar)	LPS + HA (sham)	32.6 ± 2.9	20.1 ± 6.1	19.6 ± 7.7	20.2 ± 6.0	20.6 ± 5.0	21.1 ± 5.9
	LPS + HA	27.1 ± 2.9	19 ± 2.2^†^	20.1 ± 1.1	22.5 ± 2.0	22.7 ± 2.8	23.3 ± 2.3

Oxygenation and ventilation parameters in LPS + HA (sham)- and LPS + HA-treated animals at baseline and a series of time points until the end of the experiment (data are mean ± SEM, *N* = 3–4 of each group; *P < 0.05 when compared between groups; ^†^ *P* < 0.05 when compared with baseline within per group; ^§^ *P* < 0.05 versus T0 within per group). PaO_2_, oxygen partial pressure; FiO_2_, inspiratory oxygen fraction; PaCO_2_, carbon dioxide partial pressure; P_AW_Peak, peak airway pressure; P_AW_Plateau, plateau airway pressure

**Table 4:** Systemic and pulmonary hemodynamic measurements compared between LPS + HA (sham) and LPS + HA groups

Parameter	Group	Time (h)					
		Baseline	T0	2 h	4 h	6 h	8 h
CO (l/min)	LPS + HA (sham)	6.36 ± 0.41	5.50 ± 1.98	4.86 ± 1.28	5.35 ± 1.13	5.17 ± 1.10	5.56 ± 1.27
	LPS + HA	4.98 ± 0.48	4.99 ± 0.78	5.34 ± 0.94	6.08 ± 0.97	5.05 ± 0.89	5.15 ± 0.40
MAP (mmHg)	LPS + HA (sham)	125 ± 6	82 ± 3	92 ± 20	106 ± 21	104 ± 25	96 ± 24
	LPS + HA	118 ± 7	85 ± 15	74 ± 9	86 ± 12	82 ± 17	80 ± 16
SVRI (dyn s/cm^5^/m^2^)	LPS + HA (sham)	1699 ± 59	1120 ± 154	1813 ± 688	1788 ± 468	1681 ± 354	1454 ± 248
	LPS + HA	1992 ± 156	1465 ± 423	1143 ± 231	1210 ± 347	1576 ± 778	1291 ± 461
mPAP (mmHg)	LPS + HA (sham)	23.0 ± 2.6	36.3 ± 5.6	41.3 ± 3.2	38.3 ± 5.8	38.3 ± 5.4	34.0 ± 5.1
	LPS + HA	22.7 ± 0.3	37.7 ± 5.2	44.3 ± 5.2	39.3 ± 8.0	35.3 ± 7.8	31.3 ± 10
PVRI (dyn s/cm^5^/m^2^)	LPS + HA (sham)	229 ± 31	477 ± 202	518 ± 181	467 ± 85	441 ± 92	343 ± 25
	LPS + HA	248 ± 19	404 ± 60	499 ± 72	421 ± 156	483 ± 196	416 ± 145
PAWP (mmHg)	LPS + HA (sham)	9.7 ± 2.3	12.7 ± 3.2	14.7 ± 3.0	12.0 ± 3.1	14.0 ± 4.2	13.0 ± 2.6
	LPS + HA	8.3 ± 1.2	11.0 ± 1.2	16.3 ± 1.8	13.0 ± 1.7	11.0 ± 2.0	11.7 ± 3.0
EVLWI (ml/kg)	LPS + HA (sham)	10.1 ± 3.5	13.1 ± 1.4	13.0 ± 0.6	15.9 ± 2.5	13.7 ± 1.0	12.9 ± 2.0
	LPS + HA	8.1 ± 0.3	12.4 ± 1.8	12.5 ± 1.5	13.7 ± 1.5	13.0 ± 2.1	10.1 ± 2.3
PVPI	LPS + HA (sham)	3.2 ± 1.0	4.2 ± 0.2	3.7 ± 0.5	4.4 ± 0.5	4.1 ± 0.2	4.0 ± 0.3
	LPS + HA	2.5 ± 0.2	4.1 ± 0.5	3.9 ± 1.0	3.6 ± 0.3	3.1 ± 0.7	2.9 ± 0.9

Hemodynamic parameters are shown in the LPS + HA (sham) and LPS + HA groups. Data are mean ± SEM, *N* = 3–4 of each group; CO, cardiac output; MAP, mean arterial pressure; SVRI, systemic vascular resistance index; MPAP, mean pulmonary arterial pressure; PVRI, pulmonary vascular resistance index; PAWP, pulmonary artery wedge pressure; EVLWI, extravascular lung water index; PVPI, pulmonary vascular permeability index

### Hemoadsorption blunts lung edema and histopathological signs of ARDS

Compared with LPS + HA-sham group, HA therapy for ARDS pigs caused a decrease in EVLWI, PVPI, lung wet-to-dry weight ratio and BALF cell count (Fig. 3e, f, h, i; Table 4). However, the changes did not reach statistical difference when compared with LPS + HA-sham group. But HA therapy can significantly reduce total BALF protein as compared to HA-sham treatment (Fig. 3g). These beneficial alterations were further reflected by reduced histologic signs of inflammation and injury (Fig. 3j–l).

### Hemoadsorption reduces circulating and alveolar cytokine levels

**Fig. 4:**
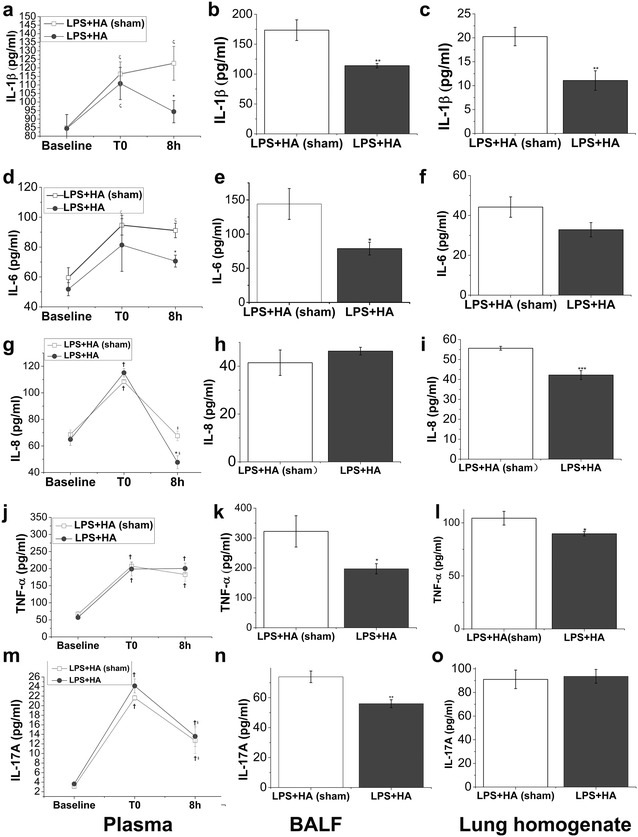
Effect of HA on the production of LPS-induced circulating and pulmonary inflammatory cytokines. Pigs were allocated into LPS + HA (sham) and LPS + HA groups. Plasma was collected at baseline, and at the time point when ALI model was diagnosed (T0) and the end of the experiment schedule (8 h after T0). BALF and lung homogenates were prepared after pigs were euthanized. The systemic and pulmonary cytokine levels of IL-1β (**a**–**c**), IL-6 (**d**–**f**), IL-8 (**g**–**i**), TNF-α (**j**–**l**) and IL-17A (**m**–**o**) were detected by using ELISA kits. Values are mean ± SEM, *N* = 4 of each group. *P < 0.05; **P < 0.01; ***P < 0.001 when compared between LPS + HA (sham) and LPS + HA groups; ^†^ *P* < 0.05 versus baseline and ^§^ *P* < 0.05 versus T0 when compared within each group

ELISA assay showed that plasma level of IL-1β and IL-6 induced by endotoxin was significantly blunted by HA treatment compared with HA-sham treatment (Fig. 4a, d). HA also elicited a remarkable decrease in the expression of IL-1β and IL-6 in both BALF and lung homogenate (Fig. 4b, c, e, f). Plasma and lung homogenate levels of IL-8 were also decreased (Fig. 4g, i). BALF levels of TNF-α and IL-17A were significantly reduced by HA treatment (Fig. 4k, n).

### Hemoadsorption-altered plasma and pulmonary proteome

**Fig. 5:**
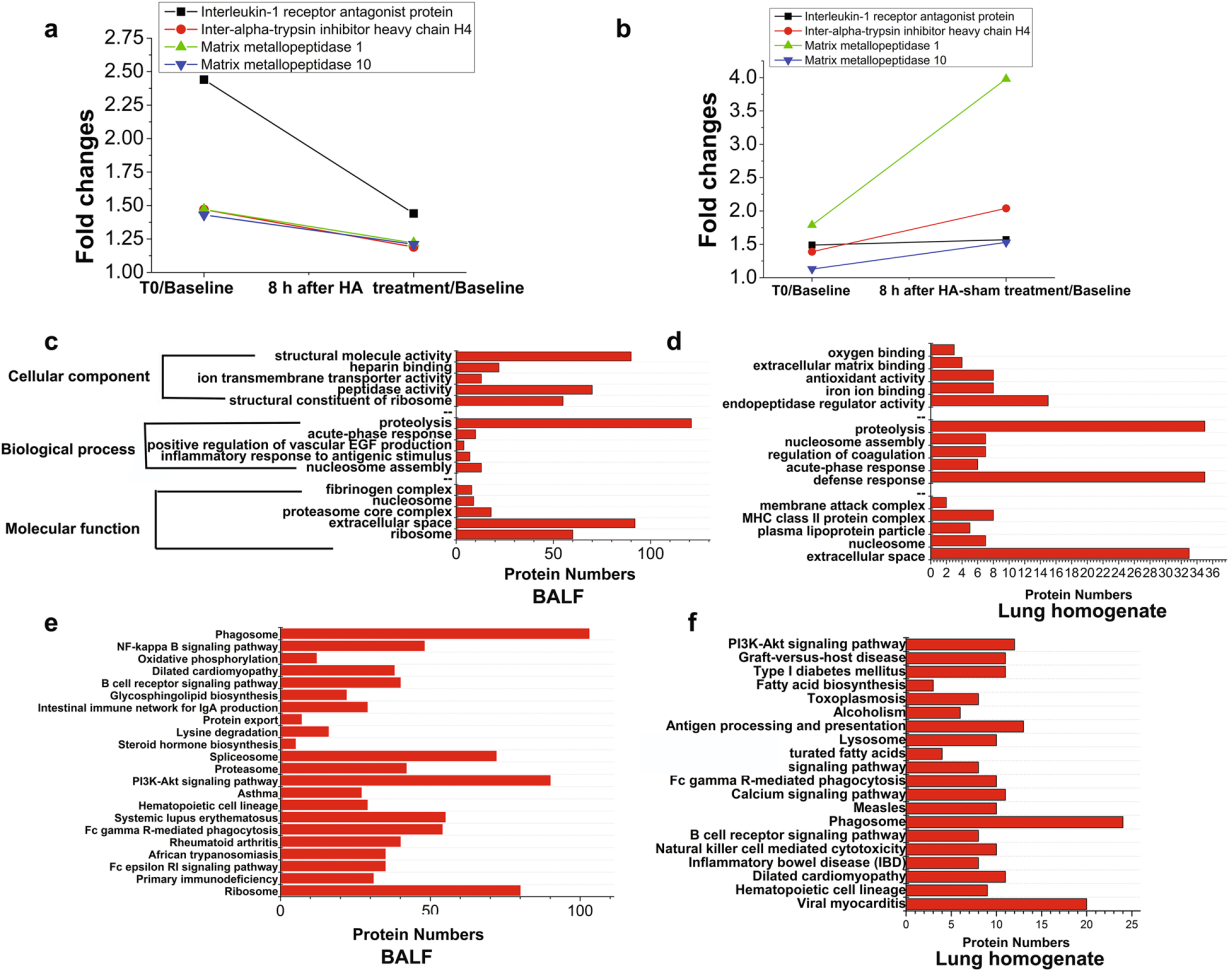
Differentially expressed plasma, BALF and lung proteins identified with iTRAQ in LPS + HA (sham)- versus LPS + HA-treated pigs. **a** and **b** four plasma proteins that were most differentially expressed after 8-h treatment in LPS + HA (sham)- versus LPS + HA-treated pigs. Note that after LPS infusion, these proteins were up-regulated (T0/baseline); however, after HA treatment, their expression were decreased. By contrast, they were further increased after HA-sham treatment. Functional annotation for differentially expressed BALF (**c**) and lung homogenate (**d**) proteins by HA and HA-sham treatment are shown. *Red bars* represent the numbers of differentially expressed proteins based on three main categories: cellular component, biological process and molecular function. Enriched KEGG pathway analysis for differentially expressed BALF (**e**) and lung homogenate (**f**) proteins by HA and HA-sham treatment are shown. *Red bars* represent the numbers of proteins in a given pathway that differentially expressed between groups

First, plasma proteome was analyzed. The differentially accumulated proteins (T0 vs baseline) and their dynamic changes after HA/HA-sham treatment (8 h after T0 vs. baseline) are shown in Additional file 5: Fig. S3. These proteins were classified into several categories based on biological process analysis (Tables 5, 6). We identified four plasma proteins , i.e., interleukin-1 receptor antagonist protein (IL-1Ra), inter-alpha-trypsin inhibitor heavy chain H4 (ITIH4), matrix metallopeptidase (MMP)-1 and MMP-10, that were up-regulated at T0 in these two groups, but sharply reduced after HA treatment while further increased in HA-sham group (Fig. 5a, b). Differentially expressed proteins (these proteins were comparable at T0 when compared with HA with HA-sham group) after 8-h HA or HA-sham treatment, and their GO annotations are also shown in Additional file 6: Table S2 and Additional file 7: Fig. S4. We then analyzed proteome in BALF and lung homogenate samples. Differentially expressed proteins compared between HA and HA-sham groups are shown in Additional file 8: Table S3, Additional file 9: Table S4, Additional file 10: Table S5, Additional file 11: Table S6. The most five relevant GO categories (*P* value less than 0.05) are shown in Fig. 5c, d. KEGG pathway analysis showed that these differentially expressed proteins were involved in the canonical signaling pathways (Fig. 5e, f).

**Table 5:** Ontology groups and associated differentially expressed plasma proteins in the time points of T0 and 8 h after HA-sham treatment versus baseline

GO biological process	Accession	Protein name	Fold change over baseline^a^	
			T0/baseline	8 h/baseline
Acute-phase response/acute inflammatory response	P79263	Inter-alpha-trypsin inhibitor heavy chain H4	1.22	2.17
	F1SH92	Inter-alpha-trypsin inhibitor heavy chain H4	1.39	2.04
	Q29056	Interleukin-1 receptor antagonist protein	1.49	1.51
	F1SFI7	Alpha-2-HS-glycoprotein (Fragment)	0.76	0.49
	Q29014	Alpha-1 acid glycoprotein (Fragment)	0.68	0.49
	Q8SPS7	Haptoglobin	0.74	0.57
	Q6S4N2	Heat shock 70 kDa protein 1B	0.64	0.65
Regulation of immune system process	P01025	Complement C3	1.45	0.88
	A5PF00	B-factor, properdin	0.75	0.62
	F1RMN7	Hemopexin	0.70	0.63
	Q69DL4	Complement C1qB (Fragment)	0.81	0.60
	A0SEH0	Complement component C6	0.71	0.76
	Q29041	Ficolin-2	0.75	0.69
Defense response/response to stress	P52552	Peroxiredoxin-2 (Fragment)	1.28	1.30
	P32195	Protegrin-2	4.76	2.43
	O02705	Heat shock protein HSP 90-alpha	1.37	2.56
	Q6QR67	Resistin	5.00	3.06
Proteolysis	C9VZX4	Matrix metallopeptidase 1	1.79	3.98
	C9VZX5	Matrix metallopeptidase 10	1.13	1.53
	F2Z528	Proteasome subunit alpha type	1.22	1.22
	F2Z5K2	Proteasome subunit alpha type	1.28	1.21
	I3LQ51	Proteasome subunit beta type	1.49	1.50
	P03974	Transitional endoplasmic reticulum ATPase	1.35	2.27
	Q9GMA6	Alpha-1-antichymotrypsin 2	0.81	0.55
	C4PGL9	Mannan-binding lectin serine peptidase 2	0.79	0.97
	F1SCF0	Alpha-1-antitrypsin	0.81	0.66
Coagulation	F1RPW2	Coagulation factor V	0.66	0.45
	F1RZ36	Coagulation factor VIII	0.64	0.74
	O97507	Coagulation factor XII	0.76	0.66
	K7GQL2	Coagulation factor XIII, A1 polypeptide	0.71	0.79
	F1RX36	Fibrinogen alpha chain	0.83	1.11
	P14460	Fibrinogen alpha chain (Fragment)	1.47	3.12
	P14477	Fibrinogen beta chain (fragment)	1.39	2.30
	P06867	Plasminogen	0.77	0.57
	F1SB81	Plasminogen	0.59	0.59
	B3STX9	Prothrombin	0.68	0.76
Negative regulation of blood coagulation	I3LRJ4	Vitamin K-dependent protein C	0.69	0.57
Metabolic process	Q29214	60S acidic ribosomal protein P0	1.47	2.45
	F1RYZ0	60S acidic ribosomal protein P2	2.17	4.72
	P01965	Hemoglobin subunit alpha	1.39	0.68
	F1RII7	Hemoglobin subunit beta	1.28	0.74
	A0SNU7	Glyceraldehyde-3-phosphate dehydrogenase (fragment)	1.23	1.41
	G9F6X8	Prolyl 4-hydroxylase beta polypeptide	1.45	2.14
	F1SN27	Sorbitol dehydrogenase	1.67	3.13
Regulation of lipid storage	Q2LE37	Apolipoprotein M	1.25	1.02
	Q29248	Apolipoprotein A-I (Fragment)	0.79	0.79
Cellular metal ion homeostasis	Q8WMN8	Lactoferrin (Fragment)	1.64	0.93
	P09571	Serotransferrin	0.77	0.63
Transport	P04404	Chromogranin-A (Fragment)	1.75	1.89
	Q29545	Inhibitor of carbonic anhydrase	0.67	0.54
	A4US67	Paraoxonase	0.75	0.67
	F1RUN2	Serum albumin	0.76	0.60
	P50390	Transthyretin	0.81	0.76
Positive regulation of cell differentiation	Q0PM28	Pigment epithelium-derived factor	0.70	0.84
Multicellular organismal process	P00761	Trypsin	0.78	0.55
Response to unfolded protein	F1RS36	78 kDa glucose-regulated protein	1.33	1.38
Cytoskeleton organization	I3LVD5	Actin, cytoplasmic 1	1.64	1.09
	A0A0B8RSX6	Filamin A, alpha	1.45	1.71
	B6VNT8	Cardiac muscle alpha actin 1	1.82	1.39
	Q6QA25	Tropomyosin 3	1.59	1.91
	P67937	Tropomyosin alpha-4 chain	1.23	1.29
	Q767L7	Tubulin beta chain	1.37	0.84
	P02543	Vimentin	1.54	5.13
Obsolete GTP catabolic process	Q0PY11	Elongation factor 1-alpha	1.35	1.37
Chromosome organization	P62802	Histone H4	2.50	1.87
Tissue remodeling	Q711S8	Secreted phosphoprotein 24	0.82	0.79
Regulation of biological process	F1S682	Sulfhydryl oxidase	0.80	0.86

^a^Note that changes are expressed as relative abundance of the plasma proteins at T0 or 8 h after HA-sham treatment compared with baseline within the LPS + HA (sham) group. A fold change ≥ 1.20, p < 0.05 represents more protein abundance in T0 or 8 h versus baseline. By contrast, a fold change ≤ 0.83, p < 0.05 represents less protein abundance in T0 or 8 h versus baseline

**Table 6:** Ontology groups and associated differentially expressed plasma proteins in the time points of T0 and 8 h after HA treatment versus baseline

GO biological process	Accession	Protein name	Fold change over baseline^a^	
			T0/baseline	8 h/baseline
Acute-phase response/acute inflammatory response	Q29014	Alpha-1 acid glycoprotein (Fragment)	2.70	0.87
	K9J6H8	Alpha-2-macroglobulin	1.82	1.01
	I3L6K3	C-reactive protein	1.43	2.50
	F1RJ76	C-reactive protein	1.27	1.32
	Q8SPS7	Haptoglobin	2.08	1.00
	P79263	Inter-alpha-trypsin inhibitor heavy chain H4	1.89	1.78
	F1SH92	Inter-alpha-trypsin inhibitor heavy chain H4	1.47	1.19
	Q29056	Interleukin-1 receptor antagonist protein	2.44	1.44
	F1S9B8	Serum amyloid A protein	1.64	1.35
Regulation of immune system process	P01025	Complement C3	1.50	1.34
	F1SMJ1	Complement component C7 (Fragment)	1.23	1.07
	A5PF00	B-factor, properdin	2.50	0.95
	F1RMN7	Hemopexin	1.85	1.08
	L8B0S2	IgG heavy chain	1.82	1.00
	L8B0W9	IgG heavy chain	1.30	0.94
	K7ZPU8	IgG heavy chain constant region (Fragment)	1.59	0.72
	Q4Z8N7	Plasma platelet-activating factor acetylhydrolase	1.20	1.38
	P28491	Calreticulin	0.81	1.29
	B0LUW3	Chemerin	0.62	0.79
	F1STZ4	Complement C1q subcomponent subunit A	0.83	0.82
	Q29041	Ficolin-2	0.77	0.72
	A5A758	Keratin 1 (Fragment)	0.53	0.69
	C4PGL9	Mannan-binding lectin serine peptidase 2	0.65	0.75
Defense response/response to stress	A2SW51	Monocyte differentiation antigen CD14	1.64	1.60
	P52552	Peroxiredoxin-2 (Fragment)	1.59	1.64
	P32195	Protegrin-2	1.25	1.05
	B3STX9	Prothrombin	1.28	1.01
	F5XVC2	von Willebrand factor	1.35	1.07
	Q6QR67	Resistin	1.79	1.52
Proteolysis	Q9GMA6	Alpha-1-antichymotrypsin 2	1.47	1.03
	F1SCF0	Alpha-1-antitrypsin	2.04	0.87
	C9VZX4	Matrix metallopeptidase 1	1.47	1.22
	C9VZX5	Matrix metallopeptidase 10	1.43	1.21
Coagulation	P14460	Fibrinogen alpha chain (Fragment)	1.54	3.30
	P14477	Fibrinogen beta chain (Fragment)	1.43	1.78
	F1RQ75	Coagulation factor IX	0.78	0.60
	F1RPW2	Coagulation factor V	0.79	0.67
	Q19AZ7	Coagulation factor VII isoform b protein	0.72	0.81
	K7GQL2	Coagulation factor XIII, A1 polypeptide	0.51	0.54
Negative regulation of blood coagulation	I3LRJ4	Vitamin K-dependent protein C	0.74	0.64
Metabolic process	F1S1G8	Amine oxidase	1.64	1.01
	P01965	Hemoglobin subunit alpha	2.17	1.11
	F1RII7	Hemoglobin subunit beta	1.89	1.22
	Q29052	Inter-alpha-trypsin inhibitor heavy chain H1	1.32	1.08
	F8SIP2	EGF-containing fibulin-like extracellular matrix protein 1	0.80	1.09
	O02668	Inter-alpha-trypsin inhibitor heavy chain H2	0.74	0.64
	I3LL80	L-lactate dehydrogenase	0.81	0.65
	Q29126	Protein WAP-3	0.31	0.84
Regulation of lipid storage	A0A0F6TNY5	APOB O	1.43	1.09
	Q29433	Apolipoprotein B (Fragment)	2.17	1.21
	Q2LE37	Apolipoprotein M	1.23	1.04
	O97674	Lipoprotein lipase (Fragment)	0.62	0.64
	D3Y264	Apolipoprotein C-II	0.80	0.63
	F1RM45	Apolipoprotein E	0.75	0.89
	Q03472	Apolipoprotein R	0.72	0.60
	Q8WMN7	Plasma phospholipid transfer protein	0.79	0.85
Cellular metal ion homeostasis	I3VKE6	Ceruloplasmin	1.30	1.07
	Q6YT39	Lactotransferrin	1.61	1.33
	P09571	Serotransferrin	2.33	0.91
	D7RK08	Transferrin receptor protein	1.45	1.30
Transport	F1RUN2	Serum albumin	2.08	1.01
	P50390	Transthyretin	1.33	0.89
Positive regulation of cell differentiation	Q0PM28	Pigment epithelium-derived factor	1.32	1.11
Multicellular organismal process	Q711S8	Secreted phosphoprotein 24	0.74	0.72
	O11780	Transforming growth factor-beta-induced protein ig-h3	0.83	0.97
	P67937	Tropomyosin alpha-4 chain	0.81	1.23
	P00761	Trypsin	0.82	0.85

^a^Note that changes are expressed as relative abundance of the plasma proteins at T0 or 8 h after HA treatment compared with baseline within the LPS + HA group. A fold change ≥1.20, *P* < 0.05 represents more protein abundance in T0 or 8 h versus baseline. By contrast, a fold change ≤0.83, *P* < 0.05 represents less protein abundance in T0 or 8 h versus baseline

## Discussion

In this current study, we tested the efficacy of “sorbent strategy”-based HA on a porcine ARDS model and found that HA reduced circulating and alveolar levels of proinflammatory cytokines, improved oxygenation and attenuated lung injuries in the exudative phase. This provides some clue that HA330 cartridge may be a novel potential weapon fighting against the “cytokine storm” on the alveolar-capillary membrane barrier.

The most commonly used large-animal models of ARDS include endotoxin infusion, repeated lavage, oleic acid and smoke/burn injury [17]. To reproduce the most known risk factor and etiology for ARDS, which is sepsis [18], we systemically administrated endotoxin (LPS) to mimic the clinically relevant sepsis-induced ARDS. The susceptibility to LPS is highly variable and differs among different animals. Pigs, sheep, calves, and cats are more sensitive to LPS challenge. Low dosage of LPS (μg/kg range) can induce significant ARDS-like features in these animals. In contrast, animals such as rodents or dogs require much higher doses to develop lung injury (mg/kg range). This different vulnerability to LPS may be induced by the pulmonary intravascular macrophages (PIM), a lung resident population of mature macrophage [19]. 8–100 μg/kg of LPS has been used in several previous studies to develop porcine ARDS model [12, 20, 21]. In our study, low amount of LPS (50ug/kg in 500 ml saline for 2 h) can also induce the pigs to develop an abrupt decrease in oxygenation and arterial pressure and a decrease in systemic vascular resistance, needing rapid fluid resuscitation (10–20 ml/kg/h of saline) and the usage of vasopressor infusion (0.5–1.5 μg/kg/min of norepinephrine).

In most recent large-animal studies, the diagnosis of experimental ARDS is ambiguous without definite criteria [12, 18, 21, 22]. To determine whether ARDS really occurred, we adopt the four diagnostic criteria released by ATS official statement on the definition of ARDS in animals [14]. ARDS model was initially diagnosed when PaO_2_/FiO_2_ is less than 200 mmHg with a PEEP at 5 cmH_2_O (criteria (a) moderate ARDS in human beings according to Berlin definition). Then, the ARDS model was further assessed with other criteria listed as follows: (b) permeability. Permeability assessment was performed by using four parameters, i.e., EVLWI, PVPI, BALF protein and the ratio of wet to dry lung weight (W/D ratio). EVLW is the amount of interstitial and alveolar fluid [23]. Previous studies showed that EVLW indexed to body weight (EVLWI) is well correlated with the value obtained by gravimetry and can independently predict mortality in septic and ARDS patients [19]. We found that both EVLWI and W/D ratio were significantly increased after LPS infusion, indicating an exudative edema phase (Fig. 1; Table 2). The permeability of lung epithelium barrier can also be calculated by PVPI [24]. PVPI in this model was increased to 5.6 ± 0.5 at T0 when ARDS was diagnosed (Fig. 1; Table 2), indicating high permeable (rather than hydrostatic) pulmonary edema because it was reported that a PVPI value of 3 can distinguish these two forms of pulmonary edema [24]. Accordingly, BALF protein concentration, another leakage marker [14], was also increased after LPS infusion (Fig. 1g). (c) Histology. To our knowledge, this is the first large-animal study to assess histologic changes at the time point that ARDS was initially diagnosed by oxygenation and ahead of therapy implementation. The major histologic findings are shown in Fig. 1j–l. It is noteworthy that LPS i.v. infusion causes only modest lung edema (perivascular edema, Additional file 4: Fig. S2) as compared to ARDS patients, because the endothelium barrier is more resistant to the damage induced by LPS [19]. (d) Inflammation. We found that both circulating and alveolar inflammatory cytokines were elevated, indicating an acute “cytokine storm” was elicited in systemic and lung milieu by i.v. infusion of LPS.

Thereafter, we conducted subsequent studies to explore the “therapeutic,” rather than “prophylactic” effect of hemoadsorption (HA) on the well-established (or far-developed, rather than “immature”) ARDS model. HA was performed with the HA330 neutral microporous resin cartridge specially designed for the absorption of medium-sized inflammatory cytokines. First, we found that HA treatment increased PaO_2_/FiO_2_ gradually (Fig. 3). Several previous studies in septic patients attributed this beneficial effect to the improved hemodynamics [10, 25]. However, a previous experimental study showed that zero-balanced high-volume continuous veno-venous hemofiltration (CVVH) could improve arterial oxygenation without systemic or pulmonary hemodynamics improvement [9]. We also found that animals treated with or without HA330-based hemadsorption did not differ in their levels of MAP, SVRI, MPAP and PVRI (Table 4). Considering that HA therapy elicited a trend in reducing lung water and the permeability of alveolar-capillary membrane barrier (Fig. 3e–i), we assume that oxygenation improvement should be partly due to the reduction of alveolar fluid leakage after clearing the peak concentration of alveolar cytokines, corresponding with the “peak concentration hypothesis” for EBP modality [26]. Lung local cytokines removal may be the result of passive spillover or active transport [26]. Notably, a recent clinical study showed that the circulating and BALF levels of IL-17A were significantly increased in ARDS patients and predicted an increased influx of alveolar neutrophils, alveolar permeability and organ dysfunction [27]. Thus, reduced BALF level of IL-17A in HA treatment group may be one of the reasons for fewer lung inflammatory cells and decreased acute lung injury. Taken together, by direct/indirect removal of a variety of pathogenic proinflammatory mediators that over-expressed in plasma and lung, HA timely blunted the “cytokine storm” in the process of ARDS and restored immunologic balance at a much lower set-point. These findings were in line with a recent small clinical study on septic-induced ARDS patients [28].

In the last step, we also examined whether HA can effect the whole proteomes in plasma and lung, which has not been previously reported. We found four plasma proteins that were most differentially regulated by HA and HA-sham therapy, i.e., IL-1Ra, MMP-1, MMP-10 and ITIH4 (Fig. 5a, b). Soluble IL-1Ra, an important member in IL-1 family of cytokines [29], was dramatically elevated in ARDS and septic patients [30, 31]. A persistent elevated level of IL-1Ra indicates immunoparalysis, which greatly contributes to the later deaths who survive the initial cytokine storm in ARDS [32]. ITIH4 is a plasma glycoprotein that belongs to a serine protease inhibitor family and acts as an acute-phase protein in several diseases [33]. However, there is a lack of studies aiming to determine the role of ITIH4 as a potential diagnostic or prognostic indicator for ARDS. The matrix metalloproteinases (MMPs) are a family of proteolytic enzymes with the capacity of degrading the extracellular matrix component, thus causing tissue damage in the pathological process [34]. Previous studies were largely focused on the BAL fluid level of MMPs (including MMP2, MMP8 and MMP9) and found an elevated expression pattern [35]. However, the circulating and BALF levels of MMP-1 (interstitial collagenase) and MMP-10 (Stromeolysin 2) in ARDS animals or patients are remained largely unknown. Collectively, we concluded a composite of plasma biomarkers with IL-1Ra, MMPs and ITIH4 may be useful to predict the severity of ARDS. Of course, this hypothesis needs further verification.

We also determined the role of HA330 cartridge-based HA on BALF and lung homogenate proteome (Additional file 8: Table S3, Additional file 9: Table S4, Additional file 10: Table S5, Additional file 11: Table S6). Notably, we found that the BALF level of secreted histone H2A, H2B and H4 was significantly blunted after HA therapy compared with HA-sham-treated pigs. Extracellular histones are the constituent of neutrophil extracellular traps (NETs) structures that ensnare and kill bacteria [36]. Elevated extracellular histones in BALF samples from humans with ARDS have been reported [37]. Also, instillation of neutralizing anti-histone H2A/H4 antibody reduced experimental ARDS severity [38]. Because extracellular histones origin from actively secretion by activated inflammatory cells, or from passively release by necrotic cells, we concluded that HA can significantly reduced the BALF level of histones by attenuating inflammatory lung injuries. BALF and lung level of Surfactant protein (SP)-B and SP-C were also significantly up-regulated by HA versus HA-sham treatment. Previous studies showed decreased levels of SP-A, SP-B and SP-C in BALF of ARDS patients [39]. Thus, HA treatment may improve oxygenation by restoring the alveolar levels of surfactant proteins. Collectively, we showed with the first experience that HA330-directed HA performance has a profound impact on plasma and lung proteome in a sepsis-induced ARDS porcine model.

Our studies have some strength. First, we comprehensively assessed the lung histologic features and other biomarkers when the ARDS model was initially diagnosed by oxygenation. Second, we first assessed the effect of HA on experimental ARDS model by using iTRAQ-labeled proteomic technology. However, our studies also have some limitations. This is a small sample animal study. Also, the effect of HA330 directed HA should be further tested by other ARDS models. Third, the experimental data cannot be directly introduced into the real clinical practice. There is still a long way to go that HA-330 cartridge-based HA can be used in ARDS patients.

## Conclusions

In conclusion, HA330 resin-based HA attenuated experimental ARDS by blunting circulating and lung “cytokine storm,” improving permeability of alveolar barrier and promoting the recovery of the disordered proteomes.

## Additional files

**Additional file 1.** Supplement-Methods.

**Additional file 2: Fig. S1.** Experimental protocol. Pigs received surgical preparation for instrumentation and divided into 4 groups. ALI was induced by intravenously infusion of LPS infusion (50 μg/kg over 2 h dissolved in 500 ml saline). Saline control group were challenged by equal amount of saline instead of LPS. When ALI was initially diagnosed (PaO_2_/FiO_2_ ≤ 200 mmHg with PEEP = 5 cmH_2_O; the time point was set as T0), pigs in LPS and saline groups were sacrificed for BAL and histology examination in order to further assess the ALI model. Other subset of pigs were subsequently treated with either HA-sham (LPS+HA-sham group), or HA (LPS+HA group) performance 3 h. Following this, the observation period was 5 h until the end of the experiment. Pigs in the 2 groups were sacrificed for BAL and histology examination. Physiological variables were recorded as indicated.

**Additional file 3: Table S1.** Lung injury scoring system.

**Additional file 4: Fig. S2.** Typical lung pathological changes induced by i.v. infusion of LPS. Representative pig lung sections stained with HE are shown. (A): Note atelectasis and the thickened alveolar walls. The majority of the alveoli are infiltrated with inflammatory cells (40×). (B): Arrow shows patchy neutrophilic infiltrates in alveolar spaces; “†**”**indicates thickened alveolar walls with septal neutrophils (400×). (C): Note the presence of deposition of pink fibrin strands (asterisk) and septal neutrophils (400×). (D): Note the perivascular edema with interstitial neutrophilic infiltrates (400×).

**Additional file 5: Fig. S3.** Dynamic expressions of plasma proteins showed with hierarchical cluster analysis. The heatmap represents the log_2_ transformed fold change for each protein indicated. Columns represent comparisons between T0/baseline and 8 h after T0/baseline in HA and HA-sham treatment group, respectively; rows represent protein accession numbers. Red colors indicate up-regulated proteins and green colors indicate down-regulated proteins, respectively.

**Additional file 6: Table S2.** Ontology groups and associated differentially expressed plasma proteins in the time points of 8 h after HA versus HA-sham treatment.

**Additional file 7: Fig. S4.** Hierarchical clustering of differentially accumulated proteins compared between HA and HA-sham treatment groups. The heatmap represents the log_2_ transformed fold change for each protein indicated. Columns represent comparisons between HA and HA-sham treatment groups at T0 and 8 h after T0, respectively; rows represent protein accession numbers. Red colors indicate up-regulated proteins and green colors indicate down-regulated proteins, respectively.

**Additional file 8: Table S3.** BALF proteins with significantly lower expression in LPS + HA versus LPS+HA (sham)-treated pigs.

**Additional file 9: Table S4.** BALF proteins with significantly higher expression in LPS + HA- versus LPS+HA (sham)-treated pigs.

**Additional file 10: Table S5.** Lung homogenate proteins with significantly lower expression in LPS+HA versus LPS + HA (sham)-treated pigs.

**Additional file 11: Table S6.** Lung homogenate proteins with significantly higher expression in LPS + HA versus LPS + HA (sham)-treated pigs.

## Abbreviations

AaDO_2_: Alveolar–arterial oxygen partial pressure difference
ARDS: Acute respiratory distress syndrome
CO: Cardiac output
CVVH: Continuous veno-venous hemofiltration
DAD: Diffuse alveolar damage
EBP: Extracorporeal blood purification
EVLW: Extravascular lung water
HA: Hemadsorption
H4, ITIH4: Inter-alpha-trypsin inhibitor heavy chain
IL-1Ra: Interleukin-1 receptor antagonist protein
PEEP: Positive end-expiratory pressure
MMP: Matrix metallopeptidase
MAP: Mean arterial pressure
MPAP: Mean pulmonary arterial pressure
NETs: Neutrophil extracellular traps
PVPI: Pulmonary vascular permeability index
PIM: Pulmonary intravascular macrophages
PVRI: Pulmonary vascular resistance index
SVRI: Systemic venous resistance index
