# Supplementary material for: Effect of HA330 resin-directed hemoadsorption on a porcine acute respiratory distress syndrome model
Source: Ann Intensive Care. 2017 Aug 14;7:84. doi: 10.1186/s13613-017-0287-0 (PMC5555961; doi:10.1186/s13613-017-0287-0)
Supplement: Supplementary file 1 — Additional file 1. Supplement-Methods. [file 13613_2017_287_MOESM1_ESM.doc]

**Supplement-Methods**

**Sedation, anesthesia and muscle relaxation**

After an overnight fasting and receiving water ad libitum, animals were first sedated with 0.25 mg/kg intramuscular (i.m.) midazolam (En Hua pharmaceutical co., Ltd., Xuzhou, JiangSu, China), and anesthetized with 2.5 mg/kg propofol (Fresenius Kabi co., Ltd., Beijing, China) injected into the auricular vein. After the animals were tied in the supine position on a heated surgical bench, anesthesia was maintained by intravenously (i.v.) injection of 8 mg/kg 3% pentobarbital sodium (Sigma-Aldrich, Shanghai, China). After the trachea was intubated, anesthesia and muscular paralysis were further maintained by continuous i.v. infusion of 8 mg/kg/h 3% pentobarbital sodium and 0.2 mg/kg/h rocuronium bromide (Organon Pharmaceutical Co., Ltd., Oss, The Netherlands).

**Ventilation and measurements of lung mechanics**

Animals were tracheostomized, intubated with an endotracheal tube (Portex, Smiths Medical International Ltd., Ashford, Kent, UK) and ventilated in a volume-controlled mode with a ventilator (Evita 4, Dräger, Lübeck, Germany). The ventilation protocol at baseline used a tidal volume of 10 ml/kg body weight, a FiO_2_ of 0.4 and a positive end-expiratory pressure (PEEP) at 0 cmH_2_O. 1 hrs after LPS infusion, PEEP was switched to 5 cmH_2_O and increased to the maximum value of 8 cmH_2_O during the procedure. When ALI was diagnosed, the tidal volume was decreased to 8 ml/kg body weight, while FiO_2_ was increased to the maximum value of 1. The ratio of inspiration period to total breathing cycle duration was 1:3 and the respiratory rate was set at 20 breaths/min throughout the experiment period. Parameters of lung mechanics, including peak airway pressure (P_aw_peak), plateau airway pressure (P_aw_plat), airway resistance and compliance were recorded at baseline and subsequent different time points.

**Instrumentation and hemodynamic measurements**

First, the right femoral artery was separated and cannulated with a thermodilution catheter (PV2015L20, Pulsion Medical Systems, Munich, Germany). Then a Swan-Ganz thermodilution catheter (774F75, Edwards Laboratories, Irvine, CA, USA) was introduced into the pulmonary artery via the right jugular vein. A 12F triple lumen catheter (Kendall, Argyle, NY, USA) was inserted into the left jugular vein and served as hemoadsorption access. A suprapubic bladder catheter (B.Braun, Melsungen, Germany) was inserted for the record of urine output. PiCCO and Swan-Ganz catheters were connected to pressure transducers, and parameters were recorded by IntelliVue MP20 monitor (Philips Healthcare, Eindhoven, the Netherlands). Measured hemodynamic variables included cardiac output (CO, the average of 3 injections of 10 ml of ice cooled saline), heart rate (HR), mean arterial pressure (MAP), systemic vascularresistance (SVR), mean pulmonary arterial pressure (MPAP), pulmonary vascular resistance (PVR), central venous pressure (CVP), pulmonary arterial wedge pressure (PAWP). Extravascular lung water (EVWL) and pulmonary vascular permeability index (PVPI) were calculated by transpulmonary thermodilution using the cold catheter. Blood gas analysis was assessed on femoral arterial and mixed venous blood samples (2 ml) using a blood gas analyzer (GEM Premier 3000, Instrumentation Laboratory, Bedford, MA, USA).

**HA330 cartridge-directed hemoadsorption**

The extracorporeal circuit consists of a dialysis machine (B.Braun), CRRT (continuous renal replacement therapy) blood lines and a hemoperfusion cartridge (HA330, Jafron Biomedical). The HA330 is an electrically neutral microporous resin that is a powerful new weapon in the clearance of “cytokine storm” occurred in sepsis . Hemoadsorption was performed from left jugular vein to left jugular vein by use of a triple-lumen catheter. Treatment was carried out for 3 hrs and the blood flow rate was set between 100-200 ml/min. Heparin was used as the systemic anticoagulant. Heparin dose for the first time should be 1 mg/kg, and the supplement dose is 8-10 mg per 30 min. The activated clotting time was maintained for 20-30 mins and the heparin injection was adjusted accordingly. HA-sham treatment was also performed to exclude the effect of extracorporeal circuit itself without hemoadsorption on the hemodynamics and lung mechanics when compared with HA-real treatment. We used a hemofilter instead of HA330 to form a closed extracorporeal circuit. Sham-treatment was carried out in the same way as for HA treatment, with the ultrafiltration line clamped.

**BALF recovery**

At the end of the experiment, a polyethylene suction catheter was inserted into the left principal bronchus and advanced into a segmental bronchus of the upper, middle and lower lobe, respectively. The catheter was connected to a 60-ml injection syringe to inject ice-cold saline and obtain serial BALF samples. Sixty milliliters of saline in three 20-ml aliquots was injected rapidly into the catheter and then withdrawn five times gently to obtain an adequate BALF samples. The pooled BALF specimens were filtrated with a 200-mesh sterile filter (Toscience Biotechnology Co., Ltd. Shanghai, China) to clear the mucus and centrifuged at 2000 rpm for 10 min at 4°C. BALF cells were re-suspended in ice-cold phosphate buffered saline (PBS) for alveolar whole white cell count by using a hemocytometer. BALF supernatant was assigned into several aliquots and frozen at -80°C for subsequent analysis.

**Assessment of total protein contents in BALF**

BCA (bicinchoninic acid) protein assay reagent kit (Pierce Biotechnology, Rockford, IL, USA) was used for the total protein determination in BALF samples according to the manufacturer’s instructions. Briefly, 25 μl of each sample was added into 200 μl working reagent, incubated at 37°C for 30 minutes in dark before reading by using a microplate reader (Tecan, Männedorf, Switzerland) at 562 nm. Values were calculated with a freshly prepared protein standard curve.

**Lung tissue collection and histopathologic evaluation**

At necropsy, the right lower lobe was excised and chopped into blocks for lung tissue sample preparation. All tissue blocks were immediately immerged into 10% neutral formalin for fixation at room temperature for at least 24 hrs. The tissues were then transferred to 70% alcohol and embedded in paraffin and cut into a series of 5-μm-thick sections. Thereafter, routine hematoxylin and eosin (H&E) staining was performed before histological assessment using light microscopy (Nikon Eclipse 55i, Melville, NY, USA). At least 20 unbiased sampling areas in each slide should be observed and independently scored at high magnification (400×total magnification) in order to minimize the regional bias. The degree of lung microscopic injury was assessed and scored based on five different histological findings according to a consensus report published previously, including the numbers of neutrophils in the alveolar space and interstitial space, the appearance of hyaline membranes and proteinaceous debris in the airspaces, and the alveolar wall thickening. Severity of lung injury was graded by a three tiered schema and the resulting injury score is a continuous value between zero and one (Table 1). Injury scores of different pigs in each group were averaged for analysis. Areas containing predominately lumens of large bronchi or blood vessels should be avoided and only slides specific to alveolar parenchyma (at least 50%) would be used for quantification.

**Concentrations of proinflammatory mediators in circulation and lung tissues**

100 mg of right lung tissue was flushed free of blood and homogenized with a hand-held homogenizer in 2 ml ice-cold PBS buffer containing 1% Triton X-100 and a protease inhibitor cocktail tablet (Roche Diagnostics GmbH, Mannheim, Germany). Debris was removed by centrifugation at 12,000 rpm at 4°C for 5 mins and the supernatant was collected. Total protein concentration was quantified by using the BCA protein assay as discussed earlier. Plasma, BALF and lung homogenate levels of IL-1β, IL-6, IL-8, TNF-α, and IL-17A, were measured by standard enzymelinked immunosorbent assay (ELISA) kits (R&D Systems, Minneapolis, MN, USA; Or Bluegene Biotech CO.,LTD, Shanghai, China) according the manufactures’ instructions.

**Plasma and lung proteome**

Equal amount of plasma from 3 pigs in HA or HA-sham treatment group was pooled together for further analysis. Plasma were collected at different time points, i.e., baseline, ALI diagnosed (T0), and the end of experiment (8 h after treatment). Protein samples of BALF and lung tissue in HA or HA-sham treatment group were collected from 3 independent pigs of each group. In order to enrich the detection of medium- and low-abundance proteins in the proteomic analysis, ProteoMiner™ Protein Enrichment Kits (Bio-Rad, Hercules, CA, USA) was used to decrease the amount of high-abundance proteins. Enriched medium and low abundance plasma/BALF/lung proteins were determined by the Bradford Protein Assay Kit and employed bovine albumin standards (Bio-Rad, Hercules, CA, USA). A total of 50 μg dissolved proteins were digested with 1μg/μL trypsin (Promega, Madison, WI, USA) at 37°C for 24 hrs. Thereafter, the digested peptides were labeled with the 8-plex iTRAQ reagents (Applied Biosystems, Foster City, CA, USA) for mass spectrometric analysis according to manufacturer's instructions. The total peptide mixture was first fractionated on a Shimadzu LC-20AB HPLC system (Kyoto, Japan) with a Phenomenex strong cation exchanger (SCX) column (Torrance, CA, USA) before separation by using liquid chromatography based-electronic spray ion (ESI)-mass spectrometry (MS)/MS analysis carried out on a Thermo fisher Q-Exactive mass spectrometer. For protein identification, MS/MS spectra were searched using MASCOT version 2.3.0 (Matrix Science, London, UK) against the Uniprot-_pig_9823 database. Protein quantification was also determined with MASCOT software by analyzing the peak intensities of every same peptide. For quantitative changes, a 1.2-fold cutoff was set to determine the differentially expressed proteins, with a *P* value less than 0.05 present in at least two replicates. Thereafter, bioinformatics analysis was performed using Gene Ontology (GO) annotation (<http://www.geneontology.org/>) and the differentially expressed proteins were categorized according to their cellular localization, biological process, and molecular function. The differentially expressed proteins were further assigned to the Kyoto Encyclopedia of Genes and Genomes (KEGG) database ([http://www](http://www/). genome.jp/kegg/pathway.html) for pathway enrichment analysis. The cluster analysis of the differentially expressed proteins was carried out using Cluster 3.0 software, and TreeView version 1.6 software was used for heatmap drawing.
