# Supplementary figures and images for: Effect of HA330 resin-directed hemoadsorption on a porcine acute respiratory distress syndrome model
Source: Ann Intensive Care. 2017 Aug 14;7:84. doi: 10.1186/s13613-017-0287-0 (PMC5555961; doi:10.1186/s13613-017-0287-0)

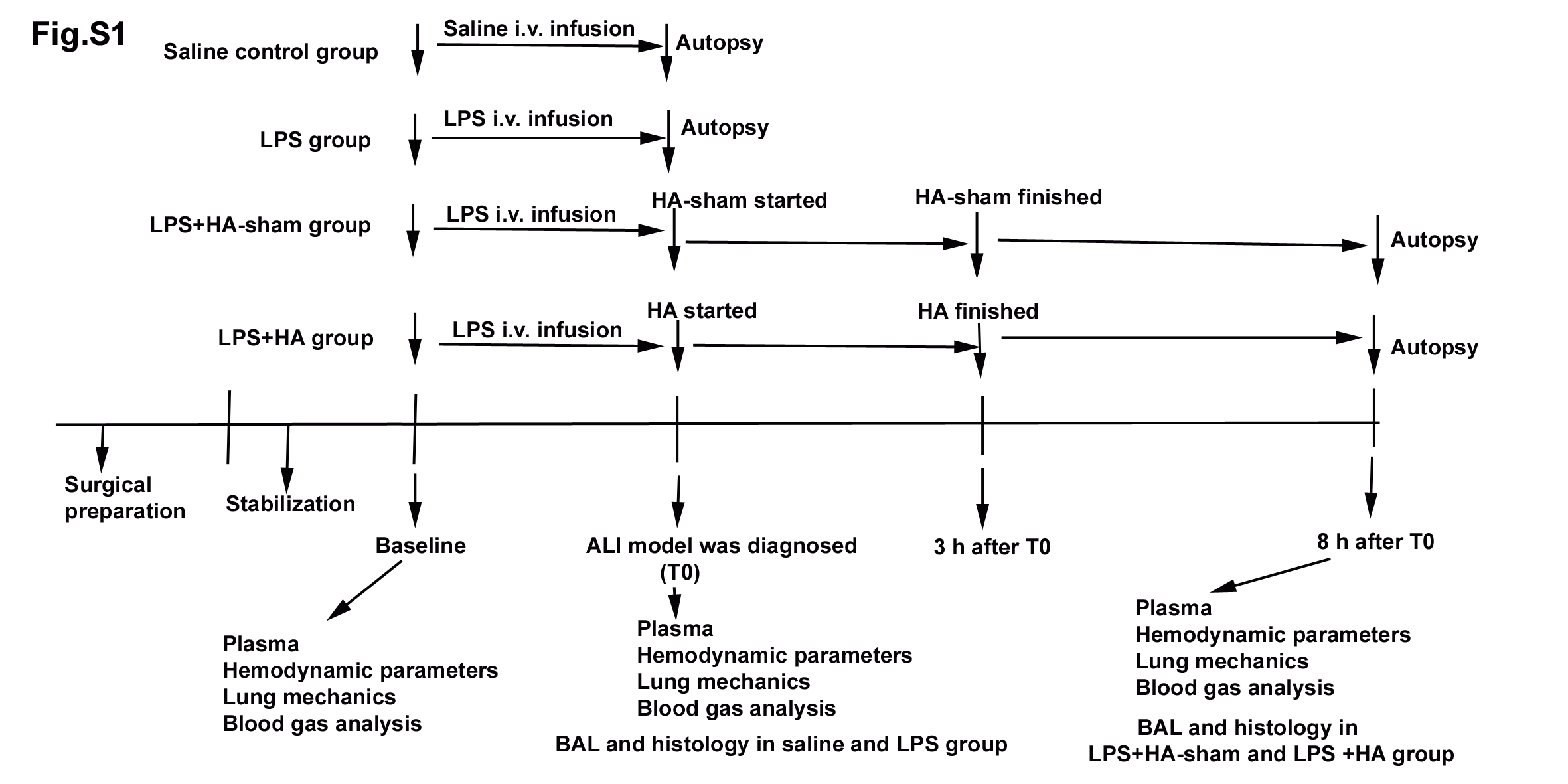

Supplement: Supplementary file 2 — Additional file 2: Fig. S1. Experimental protocol. Pigs received surgical preparation for instrumentation and divided into 4 groups. ALI was induced by intravenously infusion of LPS infusion (50 μg/kg over 2 h dissolved in 500 ml saline). Saline control group were challenged by equal amount of saline instead of LPS. When ALI was initially diagnosed (PaO2/FiO2 ≤ 200 mmHg with PEEP = 5 cmH2O; the time point was set as T0), pigs in LPS and saline groups were sacrificed for BAL and histology examination in order to further assess the ALI model. Other subset of pigs were subsequently treated with either HA-sham (LPS+HA-sham group), or HA (LPS+HA group) performance 3 h. Following this, the observation period was 5 h until the end of the experiment. Pigs in the 2 groups were sacrificed for BAL and histology examination. Physiological variables were recorded as indicated. [file 13613_2017_287_MOESM2_ESM.tif]

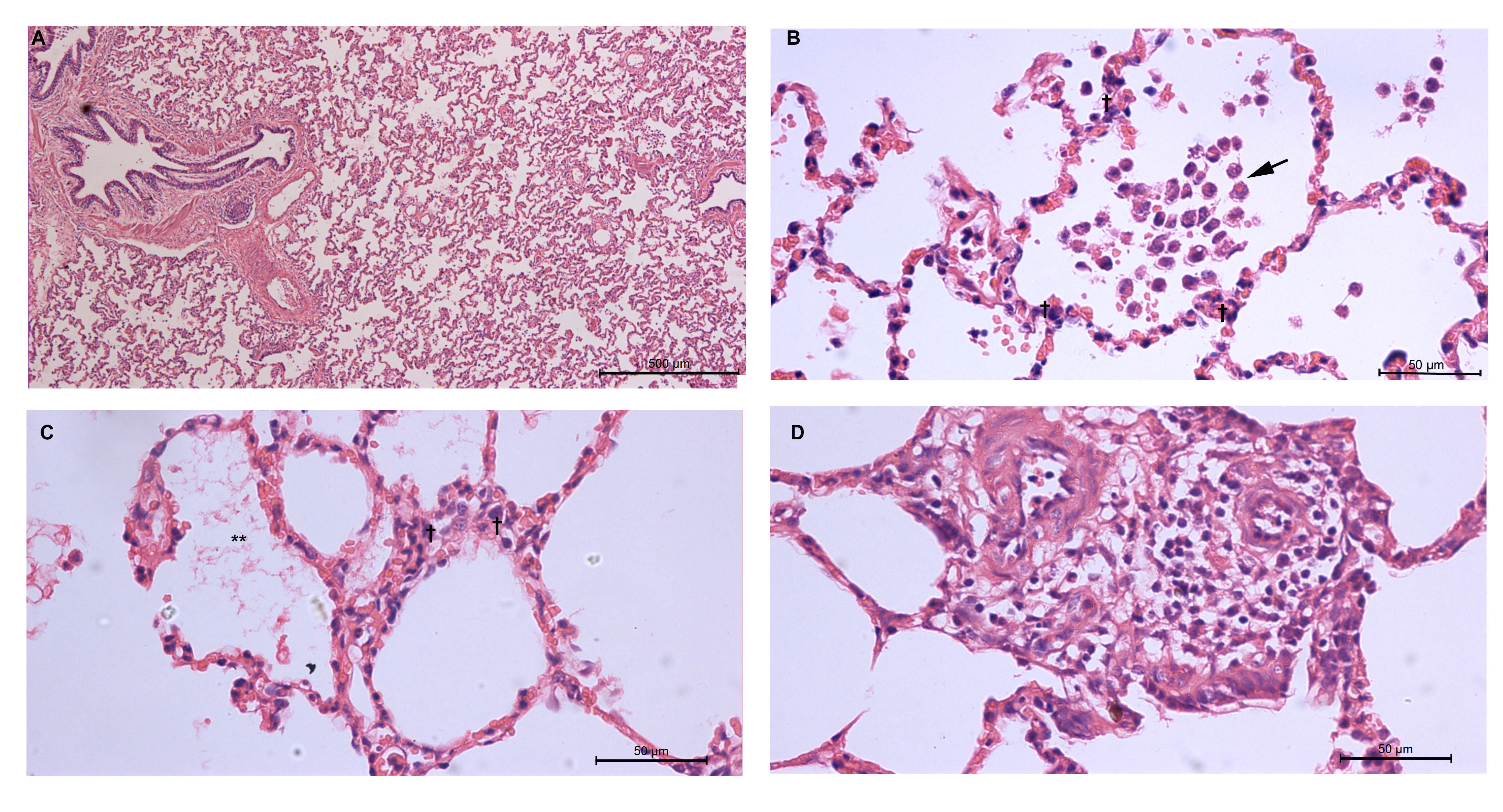

Supplement: Supplementary file 4 — Additional file 4: Fig. S2. Typical lung pathological changes induced by i.v. infusion of LPS. Representative pig lung sections stained with HE are shown. (A): Note atelectasis and the thickened alveolar walls. The majority of the alveoli are infiltrated with inflammatory cells (40×). (B): Arrow shows patchy neutrophilic infiltrates in alveolar spaces; “†”indicates thickened alveolar walls with septal neutrophils (400×). (C): Note the presence of deposition of pink fibrin strands (asterisk) and septal neutrophils (400×). (D): Note the perivascular edema with interstitial neutrophilic infiltrates (400×). [file 13613_2017_287_MOESM4_ESM.jpg]

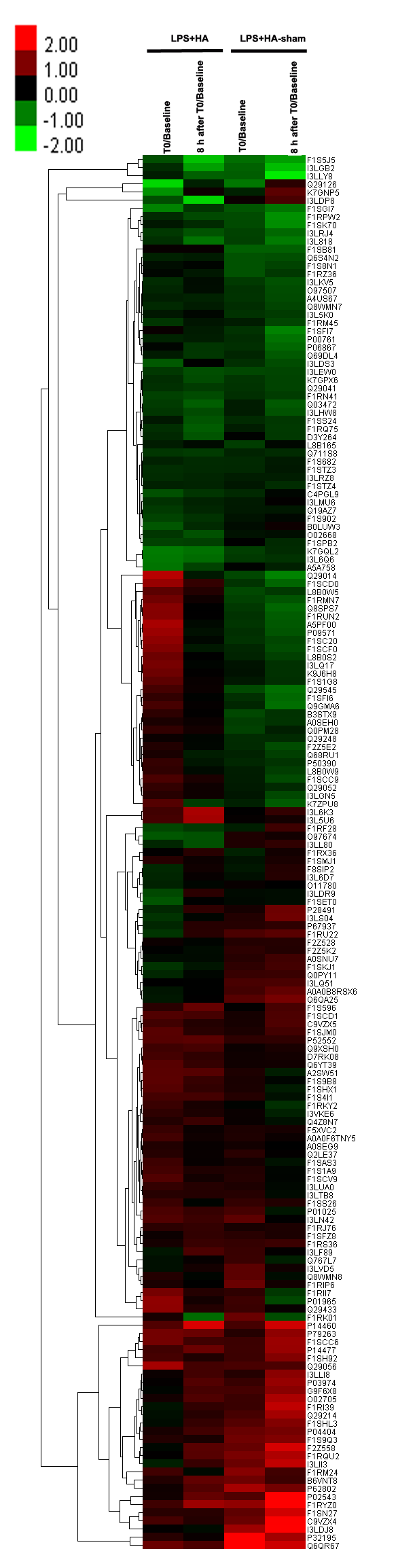

Supplement: Supplementary file 5 — Additional file 5: Fig. S3. Dynamic expressions of plasma proteins showed with hierarchical cluster analysis. The heatmap represents the log2 transformed fold change for each protein indicated. Columns represent comparisons between T0/baseline and 8 h after T0/baseline in HA and HA-sham treatment group, respectively; rows represent protein accession numbers. Red colors indicate up-regulated proteins and green colors indicate down-regulated proteins, respectively. [file 13613_2017_287_MOESM5_ESM.tif]

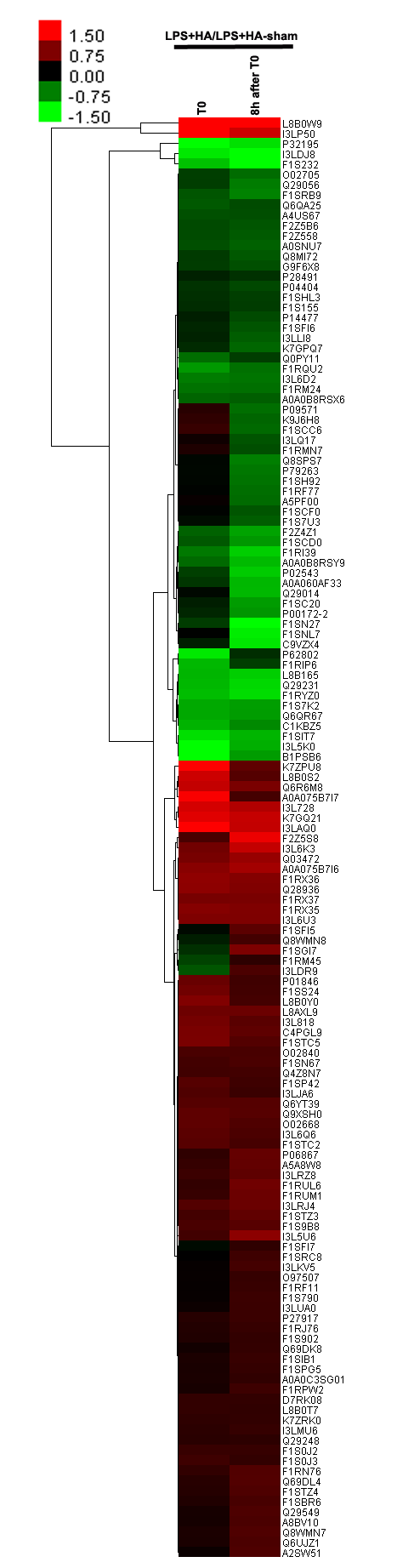

Supplement: Supplementary file 7 — Additional file 7: Fig. S4. Hierarchical clustering of differentially accumulated proteins compared between HA and HA-sham treatment groups. The heatmap represents the log2 transformed fold change for each protein indicated. Columns represent comparisons between HA and HA-sham treatment groups at T0 and 8 h after T0, respectively; rows represent protein accession numbers. Red colors indicate up-regulated proteins and green colors indicate down-regulated proteins, respectively. [file 13613_2017_287_MOESM7_ESM.tif]
