# Supplementary material for: Effect of HA330 resin-directed hemoadsorption on a porcine acute respiratory distress syndrome model
Source: Ann Intensive Care. 2017 Aug 14;7:84. doi: 10.1186/s13613-017-0287-0 (PMC5555961; doi:10.1186/s13613-017-0287-0)
Supplement: Supplementary file 3 — Additional file 3: Table S1. Lung injury scoring system. [file 13613_2017_287_MOESM3_ESM.doc]

**Table S1. Lung injury scoring system**

| Parameter | Score per field | | |
| --- | --- | --- | --- |
|  | 0 | 1 | 2 |
| A: Neutrophils in the alveolar space | none | 1–5 | ＞5 |
| B: Neutrophils in the interstitial space | none | 1–5 | ＞5 |
| C: Hyaline membranes | none | 1 | ＞1 |
| D: Proteinaceous debris filling the airspaces | none | 1 | ＞1 |
| E: Alveolar septal thickening | ＜2× | 2×-4× | ＞4× |

Score=[(20×A)+(14×B)+(7×C)+(7×D)+(2×E)]/(number of fields×100). See in Ref.12.
