# Supplementary material for: Effect of HA330 resin-directed hemoadsorption on a porcine acute respiratory distress syndrome model
Source: Ann Intensive Care. 2017 Aug 14;7:84. doi: 10.1186/s13613-017-0287-0 (PMC5555961; doi:10.1186/s13613-017-0287-0)
Supplement: Supplementary file 6 — Additional file 6: Table S2. Ontology groups and associated differentially expressed plasma proteins in the time points of 8 h after HA versus HA-sham treatment. [file 13613_2017_287_MOESM6_ESM.doc]

**Table S2 Ontology groups and associated differentially expressed plasma proteins in the time points of 8 h after HA versus HA-sham treatment**

| **GO Biological Process** | **Accession** | **Protein Name** | **8h-HA/8h-HA-sham** |
| --- | --- | --- | --- |
| Acute-phase response/ acute inflammatory response | K9J6H8 | Alpha-2-macroglobulin | 0.66 |
|  | Q29014 | Alpha-1 acid glycoprotein (Fragment) | 0.47 |
|  | Q8SPS7 | Haptoglobin | 0.59 |
|  | P79263 | Inter-alpha-trypsin inhibitor heavy chain H4 | 0.61 |
|  | F1SH92 | Inter-alpha-trypsin inhibitor heavy chain H4 | 0.62 |
|  | Q29056 | Interleukin-1 receptor antagonist protein | 0.60 |
|  | F1SFI7 | Alpha-2-HS-glycoprotein (Fragment) | 1.22 |
|  | F1RJ76 | C-reactive protein | 1.25 |
| Regulation of immune system process | A5PF00 | B-factor, properdin | 0.65 |
|  | P28491 | Calreticulin | 0.81 |
|  | F1RMN7 | Hemopexin | 0.72 |
|  | F1STZ4 | Complement C1q subcomponent subunit A | 1.39 |
|  | Q69DL4 | Complement C1qB | 1.41 |
|  | Q69DK8 | Complement C1s subcomponent | 1.23 |
|  | A2SW51 | Monocyte differentiation antigen CD14 | 1.39 |
|  |  |  |  |
| Defense response/ response to stress | B1PSB6 | Adiponectin (Fragment) | 0.53 |
|  | F1S7U3 | Chitinase-3-like protein 1 | 0.68 |
|  | P04404 | Chromogranin-A (Fragment) | 0.74 |
|  | Q6UJZ1 | Glutathione peroxidase | 1.35 |
|  | Q29549 | Clusterin | 1.37 |
|  | Q4Z8N7 | Plasma platelet-activating factor acetylhydrolase | 1.32 |
|  | F1SIB1 | Prothrombin | 1.25 |
| Proteolysis | F1SCF0 | Alpha-1-antitrypsin | 0.70 |
|  | C9VZX4 | Matrix metallopeptidase 1 | 0.39 |
| Coagulation | P14477 | Fibrinogen beta chain (Fragment) | 0.74 |
|  | F1RPW2 | Coagulation factor V | 1.28 |
|  | O97507 | Coagulation factor XII | 1.25 |
|  |  |  |  |
| Metabolic process | F1S232 | 4-trimethylaminobutyraldehyde dehydrogenase | 0.24 |
|  | F1RYZ0 | 60S acidic ribosomal protein P2 | 0.40 |
|  | A0A0B8RSY9 | Enolase 1, (Alpha) | 0.47 |
|  | F1SN27 | Sorbitol dehydrogenase | 0.35 |
|  |  |  |  |
| Regulation of lipid storage | Q29248 | Apolipoprotein A-I (Fragment) | 1.20 |
|  | P27917 | Apolipoprotein C-III | 1.27 |
|  | F1RM45 | Apolipoprotein E | 1.20 |
|  |  |  |  |
| Cellular metal ion homeostasis | Q8WMN8 | Lactoferrin (Fragment) | 1.32 |
|  | P09571 | Serotransferrin | 0.64 |
|  |  |  |  |
| Transport | I3L6D2 | Ras-related protein Rab-1A (Fragment) | 0.62 |
|  | Q8WMN7 | Plasma phospholipid transfer protein | 1.39 |
|  | D7RK08 | Transferrin receptor protein | 1.23 |
|  |  |  |  |
| cytoskeleton organization | F2Z5B6 | Tropomyosin alpha-1 chain | 0.70 |
|  |  |  |  |
| electron transport chain | P00172-2 | Isoform 2 of Cytochrome b5 | 0.54 |

*Note that the level of proteins was comparable at T0 when ALI was diagnosed and before treatment. However, they were differentially expressed following 8 hrs’ HA and HA-sham treatment respectively. Changes are expressed as relative abundance of the plasma proteins at 8 h after HA treatment compared with 8 h after HA-sham treatment. A fold change≥1.20, p＜0.05 represents more protein abundance in HA group. By contrast, a fold change ≤0.83，p＜0.05 represents less protein abundance in HA group.
