# Supplementary material for: Effect of HA330 resin-directed hemoadsorption on a porcine acute respiratory distress syndrome model
Source: Ann Intensive Care. 2017 Aug 14;7:84. doi: 10.1186/s13613-017-0287-0 (PMC5555961; doi:10.1186/s13613-017-0287-0)
Supplement: Supplementary file 8 — Additional file 8: Table S3. BALF proteins with significantly lower expression in LPS + HA versus LPS+HA (sham)-treated pigs. [file 13613_2017_287_MOESM8_ESM.doc]

**Table S3 BALF Proteins with Significantly Lower Expression in LPS+HA versus LPS+HA (sham)-treated pigs**

| **Accession** | **Protein Name** | ***P* Value** | **Fold change*** |
| --- | --- | --- | --- |
| Q56VQ1 | 2'-5' oligoadenylate synthetase 2 | 0.0048176 | 0.71 |
| F6Q5P0 | 40S ribosomal protein S13 | 2.79E-51 | 0.59 |
| P62844 | 40S ribosomal protein S15 | 0.00000739 | 0.79 |
| Q29201 | 40S ribosomal protein S16 | 1.62E-43 | 0.70 |
| Q6QAP7 | 40S ribosomal protein S17 | 2.39E-08 | 0.73 |
| P62272 | 40S ribosomal protein S18 | 3.38E-45 | 0.56 |
| A1XQU9 | 40S ribosomal protein S20 | 9.68E-16 | 0.72 |
| Q29298 | 40S ribosomal protein S23 (Fragment) | 4.67E-17 | 0.72 |
| P49171 | 40S ribosomal protein S26 | 0.000236452 | 0.70 |
| Q0Z8U2 | 40S ribosomal protein S3 | 3.38E-38 | 0.76 |
| F2Z5C7 | 40S ribosomal protein S3a | 1.27E-22 | 0.79 |
| F2Z5Q6 | 40S ribosomal protein S6 (Fragment) | 4.76E-14 | 0.74 |
| F2Z5F5 | 40S ribosomal protein S8 (Fragment) | 9.96E-39 | 0.69 |
| Q29197 | 40S ribosomal protein S9 (Fragment) | 1.65E-12 | 0.52 |
| I3LEX0 | 40S ribosomal protein S9 (Fragment) | 0.024952229 | 0.72 |
| Q29214 | 60S acidic ribosomal protein P0 | 1.39E-46 | 0.67 |
| F1RYZ0 | 60S acidic ribosomal protein P2 | 1.07E-79 | 0.62 |
| Q29195 | 60S ribosomal protein L10 | 1.03E-17 | 0.71 |
| P53027 | 60S ribosomal protein L10a (Fragment) | 5.52E-10 | 0.70 |
| I3LSD3 | 60S ribosomal protein L13 | 2.66E-17 | 0.57 |
| F1RI01 | 60S ribosomal protein L13a | 1.61E-12 | 0.78 |
| A1XQU3 | 60S ribosomal protein L14 | 0.0000513 | 0.80 |
| I3LFL4 | 60S ribosomal protein L18 (Fragment) | 1.09E-27 | 0.64 |
| P67985 | 60S ribosomal protein L22 | 1.86E-19 | 0.62 |
| P62831 | 60S ribosomal protein L23 | 4.82E-12 | 0.71 |
| A1XQU5 | 60S ribosomal protein L27 | 1.44E-14 | 0.64 |
| P62901 | 60S ribosomal protein L31 | 0.000000596 | 0.72 |
| Q6QAT0 | 60S ribosomal protein L32 | 2.6E-21 | 0.74 |
| Q29361 | 60S ribosomal protein L35 | 0.002879082 | 0.68 |
| F2Z5K6 | 60S ribosomal protein L36 | 0.00013149 | 0.72 |
| F1SJJ5 | 60S ribosomal protein L4 | 5.29E-42 | 0.72 |
| Q1HL06 | 6-phosphofructokinase (Fragment) | 0.000000652 | 0.79 |
| A5Z221 | Acetyl-CoA carboxylase alpha (Fragment) | 0.000101698 | 0.69 |
| Q6B339 | Acyl coenzyme A synthetase long-chain 1 (Fragment) | 7.23E-18 | 0.74 |
| B2CXA8 | Acyl-CoA synthetase long-chain family member 4 | 0.001230248 | 0.68 |
| A0A0B8RTX5 | Adaptor-related protein complex 2, mu 1 subunit | 0.001808368 | 0.76 |
| B9V4F0 | ADP-ribosylation factor 5 | 0.003811229 | 0.72 |
| Q007T5 | ADP-ribosylation factor 6 | 0.003438055 | 0.77 |
| Q52NJ5 | ADP-ribosylation factor-like protein 1 | 0.0000304 | 0.75 |
| I6L6E1 | Aldehyde dehydrogenase | 9.81E-19 | 0.79 |
| Q29014 | Alpha-1 acid glycoprotein (Fragment) | 2.81E-12 | 0.76 |
| F1S1G8 | Amine oxidase | 3.33E-28 | 0.70 |
| P80054 | Antibacterial protein PR-39 | 2.21E-24 | 0.55 |
| Q7M364 | Antithrombin III | 5.12E-23 | 0.74 |
| D3Y264 | Apolipoprotein C-II | 3.69E-41 | 0.69 |
| Q9TUY1 | Apolipoprotein R (Fragment) | 0.000000718 | 0.80 |
| F2Z5J4 | ATPase ASNA1 | 1.27E-08 | 0.76 |
| F1SLS7 | Autophagy-related protein 3 | 1.21E-09 | 0.76 |
| P80015 | Azurocidin | 3.97E-96 | 0.46 |
| F1S4C9 | Calcium-activated chloride channel regulator 1 | 3.04E-42 | 0.74 |
| B5L0Y4 | Calpastatin | 0.00000109 | 0.62 |
| P79322 | CAMP-regulated phosphoprotein 19k (Fragment) | 0.007779129 | 0.73 |
| O19175 | Casein kinase I isoform alpha (Fragment) | 0.02610769 | 0.79 |
| N0E654 | Casein kinase II b subunit splicing isoform 476 | 0.002447213 | 0.74 |
| F1SV36 | Caspase | 0.000276065 | 0.80 |
| D3K5L3 | Catechol-O-methyltransferase | 1.37E-13 | 0.83 |
| I1SNT7 | Catenin beta-like1 protein | 1.5E-09 | 0.53 |
| P16293 | Coagulation factor IX (Fragment) | 1.02E-127 | 0.51 |
| F1RQ75 | Coagulation factor IX | 1.77E-36 | 0.62 |
| D2SW95 | Coatomer subunit beta | 1.77E-16 | 0.76 |
| F1S982 | Coatomer subunit beta | 0.002033381 | 0.70 |
| F1SNE9 | Coatomer subunit gamma | 1.1E-25 | 0.66 |
| I3LGX5 | Collagen, type IV, alpha 3 (Goodpasture antigen) binding protein tv2 | 0.00000355 | 0.81 |
| A0SEH3 | Complement component C8G | 1.13E-21 | 0.77 |
| M3VHA2 | C-terminal binding protein 1 tv2 | 0.007145998 | 0.63 |
| F1S0P3 | C-type natriuretic peptide | 0.009516412 | 0.71 |
| Q6PUJ2 | C-X-C motif chemokine (Fragment) | 0.037387034 | 0.70 |
| G8EGP8 | C-X-C motif chemokine | 3.59E-35 | 0.58 |
| A0A0A7HF29 | DEAD (Asp-Glu-Ala-Asp) box helicase 5 | 4.12E-08 | 0.81 |
| P45846 | Dermatopontin | 5.7E-16 | 0.72 |
| Q865A4 | Double stranded RNA-dependent protein kinase | 0.008480579 | 0.76 |
| Q29125 | Elafin | 0.027481714 | 0.57 |
| G8FUN3 | Elastin (Fragment) | 0.000000726 | 0.66 |
| A0A0B8RZL6 | Eukaryotic elongation factor-2 kinase | 0.00000459 | 0.81 |
| A6M931 | Eukaryotic initiation factor 4A-III | 6.22E-08 | 0.78 |
| A0A0B8RZL4 | Eukaryotic translation initiation factor 3 subunit C | 0.000000302 | 0.82 |
| F1SKJ5 | Eukaryotic translation initiation factor 3 subunit D | 0.024560215 | 0.74 |
| A0A0B8RZM8 | Exportin-2 | 4.03E-39 | 0.76 |
| A0A0B8RTP9 | Family with sequence similarity 96, member A | 0.000997431 | 0.79 |
| I3LR69 | Ferritin | 2.18E-37 | 0.58 |
| F1RIP3 | Ferritin | 1.86E-26 | 0.63 |
| F1S6B5 | Fibromodulin (Fragment) | 0.00000388 | 0.64 |
| F1RJ25 | Fructose-bisphosphate aldolase | 0.0000338 | 0.72 |
| F1RUW7 | Gastricsin | 0.047745435 | 0.47 |
| A7UHZ6 | Gastrokine 2 | 0.001464058 | 0.61 |
| K9IW91 | GCN1 general control of amino-acid synthesis 1-like 1 | 1.51E-08 | 0.78 |
| Q8WNY0 | Glyceraldehyde-3-phosphate dehydrogenase (Fragment) | 0.0000473 | 0.65 |
| P00355 | Glyceraldehyde-3-phosphate dehydrogenase | 3.81E-166 | 0.75 |
| F1RM74 | Glyceraldehyde-3-phosphate dehydrogenase | 4.29E-16 | 0.61 |
| E0X6R2 | Glycogen synthase 1 | 0.000014 | 0.75 |
| F1SGN8 | GMP reductase | 0.003273137 | 0.80 |
| V5T860 | GTP-binding protein Rheb | 0.017022752 | 0.79 |
| Q52NJ3 | GTP-binding protein SAR1a | 0.000000199 | 0.56 |
| I7GF95 | Guanine nucleotide binding protein-like 1 | 0.0000434 | 0.74 |
| K7GKE7 | Haptoglobin | 3.08E-10 | 0. 60 |
| M3UZ46 | HEAT repeat containing 3 (Fragment) | 0.009520711 | 0.74 |
| F1RMN7 | Hemopexin | 8.51E-10 | 0.60 |
| A0A0A8IK66 | Heparin-binding protein WGA16 | 0.0000082 | 0.60 |
| Q53DY7 | Histone H1.3-like protein (Fragment) | 3.88E-26 | 0.66 |
| F2Z5P1 | Histone H2A (Fragment) | 1.19E-10 | 0.59 |
| I3L7T6 | Histone H2A | 2.05E-08 | 0.53 |
| I3LIN8 | Histone H2A | 0.00000121 | 0.70 |
| F2Z5L5 | Histone H2A | 0.0000246 | 0.41 |
| F2Z5L0 | Histone H2B | 8.91E-08 | 0.53 |
| P62802 | Histone H4 | 2.91E-119 | 0.39 |
| L8B0T7 | IgG heavy chain | 0.00012845 | 0.66 |
| I3L9F7 | Importin subunit alpha (Fragment) | 0.000746723 | 0.71 |
| Q29052 | Inter-alpha-trypsin inhibitor heavy chain H1 | 3.01E-17 | 0.82 |
| Q29056 | Interleukin-1 receptor antagonist protein | 3.83E-41 | 0.78 |
| K9IVP3 | KIAA0196 | 0.028052266 | 0.83 |
| M3VK14 | KIAA1033 | 0.031833091 | 0.69 |
| Q8WMN8 | Lactoferrin (Fragment) | 3.85E-215 | 0.62 |
| Q6YT39 | Lactotransferrin | 4.38E-24 | 0.60 |
| C7S7Z9 | Lecithin cholesterol acyltransferase | 2.18E-08 | 0.73 |
| F2Z5B1 | Leukocyte elastase inhibitor | 0.000000299 | 0.83 |
| A7J150 | Long palate lung and nasal epithelium protein 2 | 4.94E-70 | 0.48 |
| A7J153 | Long palate lung and nasal epithelium protein 5 | 1.99E-41 | 0.80 |
| A0A0B8RSK4 | Mechanistic target of rapamycin (Serine/threonine kinase) | 0.0000658 | 0.76 |
| D3K5N3 | Midkine | 0.000390466 | 0.60 |
| K9IVJ2 | Minor histocompatibility protein HA-1 | 0.011684596 | 0.74 |
| Q06AV3 | MJD1 | 0.002912552 | 0.69 |
| K9IWB5 | Nck-associated protein 1-like protein | 0.000486301 | 0.77 |
| Q68RU1 | Ovarian and testicular apolipoprotein N | 1.15E-215 | 0.65 |
| A4US67 | Paraoxonase | 3.96E-63 | 0.73 |
| P30930 | Phosphatidylcholine-sterol acyltransferase (Fragments) | 0.036212143 | 0.40 |
| Q19PY1 | Phosphorylase (Fragment) | 0.0000424 | 0.75 |
| Q9GLV6 | Probable ATP-dependent RNA helicase DDX58 | 0.000587511 | 0.70 |
| I3L7X9 | Protegrin-3 | 1.12E-109 | 0.47 |
| A0A0B8RZL2 | Protein FAM111A | 0.029336814 | 0.80 |
| Q29094 | Protein S (Fragment) | 1.08E-18 | 0.73 |
| A7E1T5 | Putative uncharacterized protein (Fragment) | 1.2E-104 | 0.76 |
| Q29582 | Pyruvate kinase M2 (Fragment) | 5.01E-12 | 0.71 |
| K9J6M1 | Ras GTPase-activating-like protein IQGAP2 | 1.24E-12 | 0.77 |
| K9J6M3 | Regulatory-associated protein of mTOR isoform 1 | 0.0000152 | 0.80 |
| E7EI20 | Rho GDP dissociation inhibitor alpha | 0.005587005 | 0.76 |
| Q06AT6 | RHOG | 6.17E-23 | 0.69 |
| I3LDZ2 | Ribonuclease 4 | 1.08E-12 | 0.74 |
| A0A0B8RZ72 | Ribosomal protein L12 | 2.03E-38 | 0.75 |
| I3LK33 | Ribosomal protein L15 | 3.62E-32 | 0.70 |
| F2Z546 | Ribosomal protein L19 (Fragment) | 3.52E-11 | 0.66 |
| B7TJ03 | Ribosomal protein L26-like 1 | 0.00000322 | 0.75 |
| A0A0B8RVJ2 | Ribosomal protein L28 | 0.000325949 | 0.76 |
| I3LFN2 | Ribosomal protein L37 | 0.002411857 | 0.81 |
| B0FWK5 | Ribosomal protein L5 | 1.13E-12 | 0.76 |
| A8YQT9 | Ribosomal protein L7 | 1.76E-15 | 0.68 |
| A0A0B8RSA9 | Ribosomal protein L7a | 7.68E-36 | 0.78 |
| F1RYZ5 | Ribosomal protein | 0.000000543 | 0.81 |
| Q711S8 | Secreted phosphoprotein 24 | 2.51E-27 | 0.59 |
| M3UZA8 | Serine/threonine kinase 3 tv1 | 0.020387094 | 0.81 |
| P09571 | Serotransferrin | 3.5E-66 | 0.50 |
| F1S9C0 | Serum amyloid A protein (Fragment) | 9.86E-43 | 0.64 |
| Q2HXZ9 | Serum amyloid A protein | 2.67E-18 | 0.74 |
| F1S9B8 | Serum amyloid A protein | 3.22E-16 | 0.70 |
| O19063 | Serum amyloid P-component | 9.87E-136 | 0.81 |
| F1S8R5 | Signal recognition particle 9 kDa protein | 0.026806504 | 0.74 |
| F1RW08 | Signal recognition particle subunit SRP68 | 1.55E-17 | 0.62 |
| G9BFT9 | Signal transducer and activator of transcription | 0.0000257 | 0.6385 |
| A1XQR9 | Small nuclear ribonucleoprotein E | 0.012457089 | 0.74 |
| I3LGQ4 | Small nuclear ribonucleoprotein Sm D2 (Fragment) | 3.84E-09 | 0.77 |
| F1S891 | Small nuclear ribonucleoprotein-associated protein | 0.000317791 | 0.81 |
| F1S682 | Sulfhydryl oxidase | 0 | 0.75 |
| Q70DY4 | TNF receptor associated factor 5 (Fragment) | 0.031191185 | 0.79 |
| I3LUD9 | Transporter (Fragment) | 0.000000113 | 0.805 |
| Q1PC32 | Triosephosphate isomerase (Fragment) | 0.038117831 | 0.78 |
| Q29220 | Tubulin alpha-3 chain (Fragment) | 0.001308743 | 0.63 |
| Q767L7 | Tubulin beta chain | 1.31E-30 | 0.69 |
| P02554 | Tubulin beta chain | 0.0000018 | 0.75 |
| Q00P21 | Tyrosine 3/tryptophan 5-monooxygenase activation protein zeta polypeptide (Fragment) | 0.015525127 | 0.76 |
| I3W8V7 | U2 small nuclear RNA auxiliary factor 2 | 0.00026229 | 0.80 |
| K9J4V0 | U5 small nuclear ribonucleoprotein 200 kDa helicase | 2.58E-12 | 0.65 |
| F1RSV2 | UDP-N-acetylglucosamine--peptide N-acetylglucosaminyltransferase 110 kDa subunit | 0.001422305 | 0.78 |
| K9J6K2 | Utrophin | 0.000261837 | 0.74 |
| P02543 | Vimentin | 1.51E-176 | 0.64 |

*Fold change is relative to LPS+HA (sham)-treated pigs, so a fold change≤0.83, p＜0.05represents less protein abundance in LPS+HA-treated pigs.
