# Supplementary material for: Effect of HA330 resin-directed hemoadsorption on a porcine acute respiratory distress syndrome model
Source: Ann Intensive Care. 2017 Aug 14;7:84. doi: 10.1186/s13613-017-0287-0 (PMC5555961; doi:10.1186/s13613-017-0287-0)
Supplement: Supplementary file 9 — Additional file 9: Table S4. BALF proteins with significantly higher expression in LPS + HA- versus LPS+HA (sham)-treated pigs. [file 13613_2017_287_MOESM9_ESM.doc]

**Table S4 BALF Proteins with Significantly Higher Expression in LPS+HA- versus LPS+HA (sham)-treated pigs**

| **Accession** | **Protein Name** | ***P* Value** | **Fold change*** |
| --- | --- | --- | --- |
| Q4GWZ2 | 40S ribosomal protein SA | 4.54E-24 | 1.47 |
| F1S232 | 4-trimethylaminobutyraldehyde dehydrogenase | 1.7E-108 | 1.54 |
| B3VMR0 | 5-aminoimidazole-4-carboxamide ribonucleotide formyltransferase/IMP cyclohydrolase | 0.0000571 | 1.25 |
| Z4YP45 | 5'-AMP-activated protein kinase subunit gamma-1 | 0.00000162 | 1.44 |
| F1RIF8 | 6-phosphogluconate dehydrogenase, decarboxylating (Fragment) | 8.99E-16 | 1.29 |
| F1RS36 | 78 kDa glucose-regulated protein | 2.9E-46 | 1.66 |
| B5APU5 | Actin related protein 2/3 complex subunit 1A | 0.000859611 | 1.44 |
| B5APU6 | Actin related protein 2/3 complex subunit 1B | 2.13E-16 | 1.53 |
| B5APU7 | Actin related protein 2/3 complex subunit 2 | 8.48E-10 | 1.62 |
| I3LVD5 | Actin, cytoplasmic 1 | 7.19E-221 | 1.43 |
| B5LX40 | Adaptor protein phosphotyrosine interaction PH domain and leucine zipper containing 1 | 0.013842498 | 1.24 |
| F1S8P9 | Adducin 1 (Alpha) | 0.000000104 | 1.34 |
| D0G0C3 | Adenosylhomocysteinase | 2.4E-43 | 1.50 |
| P00571 | Adenylate kinase isoenzyme 1 | 0.008523449 | 1.33 |
| A0A0B8S0B1 | Adenylyl cyclase-associated protein | 2.08E-82 | 1.54 |
| A0A0B8S0C3 | Adhesion regulating molecule 1 | 1.73E-11 | 1.28 |
| B1PSB6 | Adiponectin (Fragment) | 3.3E-113 | 3.01 |
| Q56P20 | ADP-ribosylation factor 4 | 0.0000291 | 1.21 |
| F1SF64 | Adseverin | 4.93E-208 | 2.87 |
| G5DGE2 | Aldo-keto reductase family 1 C1 | 7.79E-08 | 1.89 |
| Q1HE49 | Aldo-keto reductase family 1 member C4 | 1.05E-38 | 1.38 |
| Q9GKX6 | Aldose 1-epimerase | 2.11E-38 | 4.98 |
| Q5EFJ2 | Alkali myosin light chain 3 A2 catalytic | 1.89E-24 | 1.37 |
| Q8HYZ5 | Alkaline phosphatase (Fragment) | 9.66E-09 | 1.32 |
| F1SC20 | Alpha-1B-glycoprotein | 1.89E-19 | 1.39 |
| F1SFI7 | Alpha-2-HS-glycoprotein (Fragment) | 0 | 1.31 |
| E0D7H7 | Aminopeptidase T | 2.24E-213 | 1.71 |
| A5GFT5 | Aminopeptidase-like 1 | 0.01698026 | 2.96 |
| K7GLE1 | Annexin | 0.022398435 | 2.50 |
| K9IVU3 | AP-1 complex subunit gamma-1 | 2.24E-09 | 1.55 |
| F1RM45 | Apolipoprotein E | 6.27E-287 | 1.21 |
| B6DZ39 | APPL2 | 1.28E-50 | 1.39 |
| P00506 | Aspartate aminotransferase, mitochondrial | 0.000000128 | 1.88 |
| H9BYW4 | ATP citrate lyase long isoform | 1.15E-29 | 1.22 |
| F1RPS8 | ATP synthase subunit alpha heart isoform, mitochondrial (Fragment) | 2.01E-35 | 1.31 |
| Q0QEM6 | ATP synthase subunit beta (Fragment) | 5.93E-57 | 1.69 |
| Q767L0 | ATP-binding cassette sub-family F member 1 | 0.001894827 | 1.21 |
| F1S1F6 | Beclin-1 | 0.000000051 | 1.23 |
| E7D6R2 | Betaine homocysteine methyltransferase | 7.74E-39 | 1.62 |
| F1RKQ4 | Bifunctional ATP-dependent dihydroxyacetone kinase/FAD-AMP lyase (cyclizing) | 0.000000118 | 1.24 |
| K9J4N3 | Branched-chain-amino-acid aminotransferase | 0.000882627 | 1.58 |
| O02840 | Cadherin-5 | 2.04E-73 | 1.76 |
| A6YNL5 | Calpain 2 | 1.8E-53 | 1.59 |
| P04574 | Calpain small subunit 1 | 6.74E-34 | 1.39 |
| I3LQD3 | Calpain-2 catalytic subunit (Fragment) OS=Sus scrofa GN=CAPN2 PE=4 SV=1 - [I3LQD3_PIG] | 0.00000361 | 1.33 |
| Q6Q781 | Calpastatin | 1.11E-12 | 1.55 |
| P28491 | Calreticulin | 1.27E-14 | 1.59 |
| C1PIG4 | cAMP-dependent protein kinase regulatory subunit type II alpha | 0.001162441 | 1.33 |
| A0PFK5 | Capping protein (Actin filament) muscle Z-line, alpha 1 | 1.46E-23 | 1.28 |
| B6VNT8 | Cardiac muscle alpha actin 1 | 3.21E-08 | 1.40 |
| A0A0B8S0B0 | Caspase 6, apoptosis-related cysteine peptidase | 0.0000883 | 1.46 |
| F1SGS9 | Catalase | 4.08E-57 | 1.69 |
| K7GQA8 | C-C motif chemokine | 0.00000838 | 1.71 |
| A4K552 | CD45 antigen isoform 5 (Fragment) | 0.000000233 | 1.22 |
| Q007T2 | Cell division control protein 42 homolog | 0.0000164 | 1.29 |
| Q4U4N2 | Centrin 2 (Fragment) | 1.9E-09 | 1.55 |
| A0A0B8S0A2 | Chloride intracellular channel protein | 2.16E-59 | 1.23 |
| P04404 | Chromogranin-A (Fragment) | 2.72E-08 | 1.47 |
| C0MHR2 | Clathrin heavy chain | 1.63E-151 | 1.39 |
| B5ATG0 | Clathrin light chain (CLTA) protein (Fragment) | 2.71E-15 | 1.78 |
| D3K5M7 | Cleavage and polyadenylation specific factor 6 | 0.006233714 | 1.28 |
| F1RHG1 | Clustered mitochondria protein homolog | 0.000226346 | 1.24 |
| F1RRV7 | CMP-N-acetylneuraminate-beta-galactosamide-alpha-2,3-sialyltransferase 1 | 0.000025 | 1.66 |
| F1STZ4 | Complement C1q subcomponent subunit A | 1.66E-25 | 1.69 |
| Q69DK8 | Complement C1s subcomponent | 1.49E-25 | 1.36 |
| F1SBS4 | Complement C3 (Fragment) | 9.05E-19 | 2.09 |
| P01025 | Complement C3 | 3.01E-293 | 4.04 |
| F1SME1 | Complement C5a anaphylatoxin (Fragment) | 1.01E-11 | 2.88 |
| A5A8W8 | Complement component 4A | 2.27E-286 | 1.75 |
| Q6VPV1 | Complement component C5 | 3.05E-14 | 1.94 |
| Q8MI72 | Complement regulator factor H | 7.6E-47 | 1.70 |
| A7TX80 | COP9 constitutive photomorphogenic-like subunit 5 | 1.93E-11 | 1.37 |
| A7Y521 | COP9 signalosome complex subunit 4 | 0.000000129 | 1.24 |
| A7TX81 | COP9 signalosome complex subunit 6 | 0.00000384 | 1.53 |
| F1RGA9 | Coronin (Fragment) | 0.00000161 | 1.30 |
| F1RUX1 | Coronin | 2.99E-23 | 1.58 |
| F1RK53 | Coronin | 5.12E-11 | 1.55 |
| I3LPB5 | Creatine kinase B-type (Fragment) | 2.85E-51 | 4.96 |
| Q29594 | Creatine kinase B-type (Fragment) | 1.53E-09 | 2.79 |
| I3LBJ8 | Creatine kinase M-type | 2.93E-89 | 1.54 |
| A0A0B8RVZ1 | Cullin 4B | 7.67E-17 | 1.56 |
| F1RKV7 | Cytokine receptor-like factor 3 | 4.51E-15 | 1.23 |
| A5GFY8 | D-3-phosphoglycerate dehydrogenase | 0.013061224 | 1.26 |
| Q2HWR5 | Dickkopf homolog 3 | 1.63E-08 | 1.21 |
| G9F6X9 | Dihydropyrimidinase-like 2 | 0.000000592 | 1.54 |
| D3K5M4 | DIS3 mitotic control-like protein | 0.00000224 | 1.34 |
| A4F4L4 | Dynein light chain 4, axonemal | 0.00288612 | 1.27 |
| I3LD20 | Dystroglycan | 0.00237803 | 1.25 |
| Q58D68 | Ectonucleotide pyrophosphatase/phosphodiesterase family member 6 | 0.00154675 | 1.24 |
| F8SIP2 | EGF-containing fibulin-like extracellular matrix protein 1 | 2.03E-11 | 1.64 |
| Q29387 | Elongation factor 1-gamma (Fragment) | 1.27E-41 | 1.40 |
| P79381 | Epoxide hydrolase 1 | 0.00000004 | 1.58 |
| F2Z5J8 | Eukaryotic translation initiation factor 2 subunit 1 | 1.06E-27 | 1.26 |
| F8TEL5 | Eukaryotic translation initiation factor 4 gamma 1 | 4.07E-09 | 1.20 |
| A6M930 | Eukaryotic translation initiation factor 4A isoform 2 | 2.22E-23 | 1.57 |
| A0A0B8RTR5 | Extracellular matrix protein 1 | 2.01E-13 | 1.30 |
| E3UV40 | Extracellular signal-regulated kinase-2 | 1.16E-25 | 1.37 |
| D0G6X4 | Farnesyl diphosphate synthase | 0.001038242 | 1.50 |
| A0A0B8RVW6 | Farnesyltransferase, CAAX box, alpha | 0.000148444 | 1.31 |
| F8SM59 | Fatty acid synthase (Fragment) | 0.0000369 | 14.12 |
| Q28936 | Fibrinogen A-alpha-chain (Fragment) | 1.02E-59 | 2.02 |
| I3LJA6 | Fibrinogen alpha chain (Fragment) | 5.93E-08 | 1.45 |
| F1RX36 | Fibrinogen alpha chain | 4.16E-118 | 1.78 |
| Q8MIP7 | Fibrinogen-like protein 2 | 1.05E-33 | 1.27 |
| I3L5W3 | Ficolin-2 | 2.23E-41 | 1.23 |
| A0A0B8RSX6 | Filamin A, alpha | 0.000056 | 1.27 |
| O77657 | Fructose-1,6-bisphosphatase (Fragment) | 1.05E-23 | 1.94 |
| U3GT97 | Fstl1 | 0.0000045 | 2.56 |
| I3LPP1 | Fumarate hydratase, mitochondrial | 9.01E-08 | 1.55 |
| A0A0B8RSV6 | G protein pathway suppressor 1 | 6.72E-13 | 1.28 |
| B7U2G5 | Galectin | 1.54E-23 | 1.78 |
| A3EX84 | Galectin | 5.53E-27 | 1.63 |
| Q06AS6 | GBI2 | 0.011872833 | 1.34 |
| A5A779 | Geranylgeranyl transferase type-2 subunit alpha | 0.0000599 | 1.29 |
| A0A0B8RVW4 | Glucose-6-phosphate 1-dehydrogenase | 6.58E-135 | 1.36 |
| M3TYW5 | Glutamyl-prolyl-tRNA synthetase | 2.62E-09 | 1.20 |
| I3LBL7 | Glutathione S-transferase | 0.00161031 | 1.4 |
| F1S940 | Glycosyltransferase 25 domain containing 1 | 0.031452175 | 2.47 |
| B6E241 | Growth factor receptor bound protein 2 | 0.0000113 | 1.51 |
| B2XWS2 | Guanylate binding protein 2 | 0.017638825 | 1.35 |
| A4UTN7 | Heat shock 105kDa/110kDa protein 1 | 2.24E-53 | 1.48 |
| O02705 | Heat shock protein HSP 90-alpha | 0 | 4.19 |
| P01965 | Hemoglobin subunit alpha | 4.8E-48 | 1.76 |
| F1RII7 | Hemoglobin subunit beta | 0 | 1.71 |
| A0A0B8RSU5 | Hepatoma-derived growth factor | 3.97E-08 | 3.49 |
| D5KJI2 | High mobility group AT-hook protein 1 | 0.0000405 | 1.21 |
| Q9TUP1 | HnRNP A2/B1 protein (Fragment) | 0.000331653 | 1.28 |
| M3TYL1 | Hypoxia inducible factor 1, alpha subunit inhibitor | 0.003539753 | 1.34 |
| K9IWD4 | Hypoxia up-regulated protein 1 | 6.06E-08 | 1.53 |
| P01846 | Ig lambda chain C region | 0 | 1.52 |
| K7ZRK0 | IgA heavy chian constant region (Fragment) | 0 | 3.02 |
| L8AXK3 | IgG heavy chain | 7.53E-63 | 2.14 |
| L8AXM9 | IgG heavy chain | 8.05E-08 | 3.44 |
| L8B0R9 | IgG heavy chain | 4.95E-37 | 1.68 |
| L8B0S2 | IgG heavy chain | 3.41E-21 | 2.19 |
| L8B0S7 | IgG heavy chain | 0.000350766 | 2.54 |
| L8B0U3 | IgG heavy chain | 1.03E-08 | 1.88 |
| L8B0V2 | IgG heavy chain | 8.8E-29 | 1.80 |
| L8B0W0 | IgG heavy chain | 0.0000135 | 1.85 |
| L8B0W5 | IgG heavy chain | 0.012738698 | 1.36 |
| L8B0Y0 | IgG heavy chain | 0.00000166 | 1.33 |
| L8B149 | IgG heavy chain | 1.49E-29 | 1.25 |
| L8B165 | IgG heavy chain | 5.83E-104 | 2.76 |
| L8B180 | IgG heavy chain | 2.26E-08 | 3.84 |
| K7ZPU8 | IgG heavy chian constant region (Fragment) | 5.18E-15 | 2.67 |
| F1SKI5 | Inosine-5'-monophosphate dehydrogenase | 0.000000375 | 1.22 |
| A0A0B8RVT0 | Inositol-3-phosphate synthase 1 | 4.72E-10 | 1.34 |
| A9YTX9 | Interferon regulatory factor 3 | 2.94E-09 | 1.56 |
| I3LDC7 | Isocitrate dehydrogenase [NADP] (Fragment) | 9.15E-130 | 1.49 |
| P00172-2 | Isoform 2 of Cytochrome b5 | 0.0000014 | 1.35 |
| Q2VL90-2 | Isoform 2 of Scavenger receptor cysteine-rich type 1 protein M130 | 7.14E-16 | 1.62 |
| Q29116-2 | Isoform Major of Tenascin | 1.54E-29 | 2.08 |
| A5A759 | Keratin 2A | 0.0166506 | 1.45 |
| M1FV56 | Leucine-rich repeat interacting protein-1 | 0.0000557 | 1.44 |
| A0A0B8RVR2 | Leupaxin | 0.000390516 | 1.22 |
| F1SFK9 | LIM and cysteine-rich domains protein 1 | 0.000250921 | 1.38 |
| M3VJZ7 | LIM and SH3 protein 1 | 5.46E-09 | 3.09 |
| P00336 | L-lactate dehydrogenase B chain | 4.57E-170 | 1.65 |
| A0A0B8RTI4 | LPS-responsive vesicle trafficking, beach and anchor containing | 0.00000868 | 1.21 |
| A5A8V8 | LSM2 homolog, U6 small nuclear RNA associated (S. cerevisiae) | 0.001453355 | 1.64 |
| Q9N1X3 | Lung surfactant protein A (Fragment) | 0.000159586 | 1.79 |
| A0A0B8S055 | Lymphocyte-specific protein 1 | 0.000680502 | 1.58 |
| F1S458 | Lysine--tRNA ligase | 3.73E-11 | 1.46 |
| Q6EEI7 | Mannose receptor C1 (Fragment) | 0.000426873 | 1.70 |
| A0A0B8RSM0 | Metallo-beta-lactamase domain containing 1 | 0.029138905 | 3.08 |
| F1SGT2 | Methylthioribulose-1-phosphate dehydratase | 0.006283759 | 1.30 |
| B1A9N7 | MHC class I antigen | 7.24E-10 | 1.26 |
| D5K8A2 | Mitochondria-eating protein | 0.000000349 | 1.77 |
| G9F6X6 | Mitochondrial heat shock 60 kDa protein 1 | 5.05E-23 | 1.29 |
| M3TYR4 | Mitogen-activated protein kinase kinase 3 | 0.000000359 | 1.21 |
| F1SS62 | Myosin-1 (Fragment) | 0.002311277 | 1.24 |
| I3LD86 | N-acetylmuramoyl-L-alanine amidase | 1.7E-13 | 1.70 |
| A0A0B8RSJ6 | NEDD8 activating enzyme E1 subunit 1 | 1.62E-08 | 1.55 |
| Q06AT1 | Neuron-specific calcium-binding protein hippocalcin | 0.00324307 | 1.54 |
| I3LNH3 | Neutral alpha-glucosidase AB | 3.09E-35 | 1.42 |
| Q29271 | Orf protein (Fragment) | 0.0000658 | 1.30 |
| Q8MJ49 | Osteoclast-stimulating factor 1 | 0.008921451 | 1.31 |
| C5H0C6 | OTUB1 | 1.12E-16 | 1.73 |
| I3LEQ0 | Oxysterol-binding protein | 0.029964058 | 1.24 |
| M3UZB4 | Paxillin O | 0.002421272 | 1.30 |
| Q864B5 | PDJA1 chaperone | 1.7E-12 | 1.45 |
| T1RTP3 | PDZ and LIM domain protein 1 | 1.26E-08 | 1.41 |
| D3K5K1 | Periostin, osteoblast specific factor | 1.28E-08 | 1.59 |
| F1SDX9 | Peroxiredoxin-2 | 7.69E-127 | 3.01 |
| Q9TSX9 | Peroxiredoxin-6 | 3.68E-58 | 1.39 |
| A0A0B8RVL7 | Phosphatidylinositol-5-phosphate 4-kinase, type II, gamma | 0.000112064 | 1.43 |
| F1RQQ8 | Phosphorylase | 4.07E-10 | 1.20 |
| P06867 | Plasminogen | 1.08E-29 | 2.63 |
| F2Z521 | Platelet-activating factor acetylhydrolase IB subunit alpha | 3.48E-34 | 2.76 |
| G9F6X8 | Prolyl 4-hydroxylase beta polypeptide | 1.75E-43 | 4.14 |
| Q863Z0 | Proteasome activator complex subunit 2 | 7.64E-26 | 1.25 |
| P61291 | Proteasome activator complex subunit 3 | 0.000000565 | 1.53 |
| Q71M46 | Proteasome activator PA28 alpha subunit (Fragment) | 6.12E-48 | 1.32 |
| F1SSL6 | Proteasome subunit alpha type | 2.38E-27 | 1.51 |
| F2Z5L7 | Proteasome subunit alpha type | 4.22E-69 | 2.16 |
| I3LAB6 | Proteasome subunit alpha type | 1.6E-72 | 2.29 |
| F2Z528 | Proteasome subunit alpha type | 8.51E-68 | 2.69 |
| F2Z5K2 | Proteasome subunit alpha type | 3.52E-86 | 2.00 |
| F2Z5N0 | Proteasome subunit alpha type | 1.2E-90 | 2.56 |
| F1SBA5 | Proteasome subunit alpha type | 0.000111259 | 1.57 |
| A0A0B8RVK4 | Proteasome subunit beta type | 5.93E-16 | 2.51 |
| Q9TV82 | Proteasome subunit beta type | 1.21E-11 | 1.76 |
| F1S9C9 | Proteasome subunit beta type | 3.29E-14 | 1.60 |
| Q2PYM7 | Proteasome subunit beta type | 1.99E-11 | 1.81 |
| I3LQ51 | Proteasome subunit beta type | 8.35E-41 | 2.68 |
| Q09YA9 | Proteasome subunit beta type | 5.87E-12 | 2.22 |
| F1ST02 | Proteasome subunit beta type | 1.57E-58 | 1.82 |
| G3DRF8 | Proteasome subunit beta type | 6.24E-26 | 2.10 |
| A5D9J6 | Proteasome subunit beta type | 2.51E-11 | 1.58 |
| A1XQU1 | Proteasome subunit beta type-7 | 7.24E-24 | 1.9 |
| A0A0B8RVS2 | Proteasome-associated protein ECM29-like protein | 0.032782547 | 1.37 |
| E1CAJ6 | Protein disulfide isomerase P5 | 1.67E-25 | 1.62 |
| A0A0B8RZ91 | Protein phosphatase 1, regulatory (Inhibitor) subunit 2 | 0.00000536 | 1.52 |
| I3LJT9 | Protein phosphatase methylesterase 1 (Fragment) | 0.00000401 | 1.84 |
| Q2EN75 | Protein S100-A6 | 0.014851435 | 1.37 |
| P50118 | Proto-oncogene serine/threonine-protein kinase mos | 6.41E-08 | 1.56 |
| F1SVC0 | Pulmonary surfactant-associated protein B | 6.2E-79 | 1.65 |
| Q9N1X4 | Pulmonary surfactant-associated protein D | 1.02E-13 | 1.29 |
| F1S8H8 | Purine nucleoside phosphorylase (Fragment) | 0.000000325 | 1.93 |
| K7ZMG1 | Putative cysteine-rich protein 2 | 9.9E-13 | 1.57 |
| O46560 | Pyridoxal kinase | 2.7E-19 | 1.61 |
| K7GLA7 | Pyruvate dehydrogenase E1 component subunit alpha, somatic form, mitochondrial | 0.000000544 | 1.33 |
| F1SV14 | Radixin | 0.00000119 | 1.26 |
| K9IWF9 | Ras GTPase-activating-like protein IQGAP1 | 0 | 1.42 |
| K9J4Q7 | Rho GTPase-activating protein 35 | 0.025492179 | 1.20 |
| P10775 | Ribonuclease inhibitor | 7.27E-18 | 1.38 |
| D0G777 | S-adenosylmethionine synthase | 9.1E-23 | 1.66 |
| F1SU97 | Saposin-B-Val | 0.001089523 | 3.19 |
| A6N9J9 | Secreted phosphoprotein 1 | 3.8E-10 | 13.03 |
| Q1L128 | Secretoglobin family 1A member 1 (Fragment) | 1.05E-39 | 2.39 |
| Q3YLA6 | Serine/arginine-rich splicing factor 1 | 8.33E-13 | 1.82 |
| Q06A98 | Serine/arginine-rich splicing factor 2 | 1.65E-34 | 1.93 |
| A0A0B8RVG3 | Serine/threonine kinase 10 | 1.26E-09 | 1.28 |
| Q06AA0 | SFRS6 | 2E-17 | 1.88 |
| B8XH67 | Solute carrier family 9 (Sodium/hydrogen exchanger) member 3 regulator 1 | 0.00257866 | 1.35 |
| A3RIE0 | SPARCL-1 | 2.23E-08 | 3.08 |
| A0A0B8RZ36 | Spectrin, alpha, non-erythrocytic 1 | 9.4E-38 | 1.358 |
| A0A0B8RZ38 | Sperm associated antigen 9 | 4.91E-13 | 1.23 |
| F1S7B1 | Sperm surface protein Sp17 | 0.001050618 | 2.66 |
| A0A0B8RZS7 | Sterol O-acyltransferase 2 | 3.99E-08 | 1.50 |
| F1SFR6 | Succinyl-CoA ligase [GDP-forming] subunit beta, mitochondrial | 0.000928157 | 1.25 |
| I3LRP8 | Sulfotransferase (Fragment) | 2.84E-37 | 1.62 |
| Q95MF8 | Sulfotransferase | 1.02E-34 | 1.72 |
| A7MAK4 | Surfactant protein B | 8.95E-47 | 2.62 |
| F1SQN1 | T-complex protein 1 subunit delta | 1.06E-131 | 1.21 |
| Q29068 | T-complex protein 1 subunit gamma (Fragment) | 0.0000177 | 1.28 |
| B6CVD6 | Thioredoxin domain-containing 4 | 0.000000108 | 1.20 |
| D7RK08 | Transferrin receptor protein | 0.004719151 | 1.29 |
| O11780 | Transforming growth factor-beta-induced protein ig-h3 | 0.00012003 | 1.24 |
| A0A0B8RZZ6 | Transglutaminase 2 | 9.47E-19 | 1.50 |
| A8U4R4 | Transketolase | 0 | 2.47 |
| Q6QA25 | Tropomyosin 3 | 2.15E-25 | 1.57 |
| K9IVV5 | Tryptophan--tRNA ligase, cytoplasmic isoform a | 7.37E-20 | 1.39 |
| P80220 | TSC22 domain family protein 3 | 0.034313404 | 2.03 |
| I3LRU5 | Ubiquitin carboxyl-terminal hydrolase (Fragment) | 1.67E-10 | 1.22 |
| B6DT15 | Ubiquitin carboxyl-terminal hydrolase | 0.000162705 | 1.53 |
| A0A0B8RZ10 | Ubiquitin-like modifier activating enzyme 1 | 1.76E-35 | 1.39 |
| Q9N0Y9 | Ubiquitous tropomodulin U-Tmod | 9.02E-12 | 1.37 |
| K9J4P3 | UDP-glucose glycoprotein glucosyltransferase 1 | 0.014211993 | 1.27 |
| I3LBB2 | Vacuolar protein sorting-associated protein 35 (Fragment) | 8.37E-27 | 1.31 |
| K7GNN0 | von Willebrand factor | 5.12E-08 | 1.79 |
| K9IWA3 | V-type proton ATPase subunit S1 | 1.26E-11 | 1.21 |
| M3TYU5 | WD repeat-containing protein 44 isoform 1 | 0.00005 | 1.46 |
| Q19QU3 | Zinc finger Ran-binding domain-containing protein 2 | 0.009285666 | 1.25 |

*Fold change is relative to LPS+HA(sham)-treated pigs, so a fold change≥1.2, p＜0.05 represents increased protein abudance in LPS+HA -treatment pigs.
