# Supplementary material for: Effect of HA330 resin-directed hemoadsorption on a porcine acute respiratory distress syndrome model
Source: Ann Intensive Care. 2017 Aug 14;7:84. doi: 10.1186/s13613-017-0287-0 (PMC5555961; doi:10.1186/s13613-017-0287-0)
Supplement: Supplementary file 10 — Additional file 10: Table S5. Lung homogenate proteins with significantly lower expression in LPS+HA versus LPS + HA (sham)-treated pigs. [file 13613_2017_287_MOESM10_ESM.doc]

**Table S5 Lung Homogenate Proteins with Significantly Lower Expression in LPS+HA versus LPS+HA (sham)-treated pigs**

| **Accession** | **Protein Name** | ***P* Value** | **Fold change*** |
| --- | --- | --- | --- |
| A5Z221 | Acetyl-CoA carboxylase alpha (Fragment) | 0.000274922 | 0.80 |
| Q29014 | Alpha-1 acid glycoprotein (Fragment) | 4.26E-13 | 0.66 |
| Q9GMA6 | Alpha-1-antichymotrypsin 2 | 2.34E-07 | 0.79 |
| F1SCF0 | Alpha-1-antitrypsin | 7.75E-35 | 0.80 |
| F1SC20 | Alpha-1B-glycoprotein | 1.05E-27 | 0.72 |
| F1SFI7 | Alpha-2-HS-glycoprotein (Fragment) | 9.24E-61 | 0.56 |
| P80054 | Antibacterial protein PR-39 | 2.29E-09 | 0.75 |
| F1SJT7 | Apolipoprotein A-IV | 2.46E-09 | 0.64 |
| P27917 | Apolipoprotein C-III | 7.71E-11 | 0.767 |
| A0A0B8RTW8 | BCL2-associated athanogene 3 | 1.21E-07 | 0.80 |
| A5PF00 | B-factor, properdin | 1.29E-21 | 0.74 |
| E5Q8C6 | CCAAT enhancer binding protein beta | 0.00235083 | 0.66 |
| F1SMJ1 | Complement component C7 (Fragment) | 1.60E-07 | 0.82 |
| Q8MI72 | Complement regulator factor H | 0.008016014 | 0.70 |
| F1RRW2 | Cytochrome b561 | 0.005468995 | 0.81 |
| Q8SQ69 | Cytochrome P450 1A1 G | 2.34E-08 | 0.64 |
| A3EX84 | Galectin | 3.26E-31 | 0.83 |
| J9JIK0 | Glutathione peroxidase (Fragment) | 2.24E-09 | 0.76 |
| L8B0T7 | IgG heavy chain | 0.01362263 | 0.75 |
| Q29545 | Inhibitor of carbonic anhydrase | 0.042756945 | 0.80 |
| Q7YQ92 | MHC class II antigen (Fragment) | 1.20E-11 | 0.78 |
| A0A077EVQ4 | NADH-ubiquinone oxidoreductase chain 4 | 0.04151698 | 0.69 |
| F1SB81 | Plasminogen | 0.000388734 | 0.76 |
| F1RLQ2 | Prelamin-A/C | 6.39E-37 | 0.73 |
| P49933 | Protegrin-4 | 0.040232544 | 0.72 |
| P04366 | Protein AMBP (Fragment) | 9.47E-06 | 0.69 |
| F1SIB1 | Prothrombin | 7.92E-08 | 0.74 |
| F1SC80 | Retinol-binding protein 4 | 0.001315738 | 0.60 |
| P09571 | Serotransferrin | 5.53E-138 | 0.70 |
| F1RUN2 | Serum albumin | 0 | 0.66 |
| B9P414 | Serum amyloid A protein (Fragment) | 0.013367499 | 0.68 |
| V5PZU4 | Testis-specific serine kinase 4 isoform 1 | 2.37E-05 | 0.82 |
| P50390 | Transthyretin | 4.47E-14 | 0.63 |
| Q29554 | Trifunctional enzyme subunit alpha, mitochondrial | 1.01E-12 | 0.80 |
| I3L638 | Vitronectin | 2.93E-08 | 0.76 |

*Fold change is relative to LPS+HA (sham)-treated pigs, so a fold change≤0.83 represents less protein abundance in LPS+HA-treatment pigs.
