# Supplementary material for: Effect of HA330 resin-directed hemoadsorption on a porcine acute respiratory distress syndrome model
Source: Ann Intensive Care. 2017 Aug 14;7:84. doi: 10.1186/s13613-017-0287-0 (PMC5555961; doi:10.1186/s13613-017-0287-0)
Supplement: Supplementary file 11 — Additional file 11: Table S6. Lung homogenate proteins with significantly higher expression in LPS + HA versus LPS + HA (sham)-treated pigs. [file 13613_2017_287_MOESM11_ESM.doc]

**Table S6 Lung homogenate Proteins with Significantly higher Expression in LPS+HA versus LPS+HA (sham)-treated pigs**

| **Accession** | **Protein Name** | ***P* Value** | **Fold change*** |
| --- | --- | --- | --- |
| I3LEX0 | 40S ribosomal protein S9 (Fragment) | 0.009284812 | 1.21 |
| P62901 | 60S ribosomal protein L31 | 0.000226187 | 1.22 |
| F1SJJ5 | 60S ribosomal protein L4 | 3.13E-25 | 1.20 |
| B5APU8 | Actin related protein 2/3 complex subunit 3 | 7.40E-09 | 1.20 |
| I3LVD5 | Actin, cytoplasmic 1 | 2.34E-68 | 1.21 |
| D3K5K4 | Acyl-CoA synthetase long-chain family member 5 | 5.35E-26 | 1.24 |
| Q6QRN9 | ADP/ATP translocase 3 | 7.28E-05 | 1.24 |
| Q9GK83 | Allograft inflammatory factor-1 (Fragment) | 9.08E-11 | 1.363 |
| Q29075 | Antimicrobial peptide NK-lysin (Fragment) | 0.000813352 | 1.26 |
| K9IVU3 | AP-1 complex subunit gamma-1 | 0.000506526 | 1.24 |
| Q95282 | ATP synthase subunit 9, mitochondrial (Fragment) | 7.83E-05 | 2.18 |
| K9IVN2 | ATP-binding cassette sub-family A member 3 | 4.74E-14 | 1.31 |
| Q00P28 | Beta-2-microglobulin protein (Fragment) | 8.49E-21 | 1.24 |
| Q4U1U3 | Cathepsin D | 1.52E-21 | 1.23 |
| Q5MJE5 | Cathepsin D protein (Fragment) | 8.52E-28 | 1.25 |
| A0A0B8S098 | Cathepsin S | 1.18E-31 | 1.32 |
| A5GFX7 | Cathepsin Z | 9.66E-08 | 1.24 |
| A7TX80 | COP9 constitutive photomorphogenic-like subunit 5 | 3.56E-06 | 1.33 |
| I3LER5 | Cytochrome c oxidase subunit 4 isoform 1, mitochondrial | 2.44E-08 | 1.21 |
| P22411 | Dipeptidyl peptidase 4 | 5.01E-21 | 1.28 |
| A0A0B8RTR8 | Dopey family member 2 | 2.46E-06 | 1.34 |
| K9IVL3 | Egf-like module containing, mucin-like, hormone receptor-like 1 OS=Sus scrofa GN=EMR1 PE=2 SV=1 - [K9IVL3_PIG] | 0.003330115 | 1.38 |
| F2Z5J5 | Enhancer of rudimentary homolog | 0.032502335 | 1.21 |
| F1S1X4 | Ferrochelatase (Fragment) | 1.61E-06 | 1.62 |
| B7U2G5 | Galectin | 0.008161586 | 1.51 |
| Q6QAS1 | Glutaredoxin (Fragment) | 1.09E-45 | 1.35 |
| Q000H8 | Glutathione S-transferase mu 2 (Fragment) | 2.50E-08 | 1.59 |
| A5A766 | Glycoprotein NMB | 8.81E-38 | 1.42 |
| K9IVM5 | GTPase IMAP family member 4 | 7.28E-31 | 1.33 |
| Q8SPS7 | Haptoglobin | 1.21E-126 | 1.223 |
| P02067 | Hemoglobin subunit beta | 6.27E-09 | 1.23 |
| F1RII7 | Hemoglobin subunit beta | 0 | 1.40 |
| Q4TTS4 | Histone H1.2-like protein | 1.01E-08 | 1.23 |
| F2Z5L5 | Histone H2A | 0.000483941 | 1.27 |
| F2Z5L0 | Histone H2B | 0.000342269 | 1.47 |
| F2Z576 | Histone H3 | 4.01E-23 | 1.27 |
| P62802 | Histone H4 | 1.36E-218 | 1.32 |
| B2ZF49 | Hydroxyacyl-coenzyme A dehydrogenase/3-ketoacyl-coenzyme A thiolase/enoyl-coenzyme A hydratase alpha subunit | 0.001217172 | 1.55 |
| P01846 | Ig lambda chain C region | 0 | 1.43 |
| K7ZRK0 | IgA heavy chian constant region (Fragment) | 0 | 2.58 |
| L8B0R9 | IgG heavy chain | 1.73E-10 | 1.34 |
| L8B0S2 | IgG heavy chain | 2.16E-20 | 1.38 |
| L8B0W9 | IgG heavy chain | 7.73E-65 | 1.53 |
| L8B149 | IgG heavy chain | 1.10E-30 | 1.61 |
| K7ZPU8 | IgG heavy chian constant region (Fragment) | 1.52E-09 | 1.61 |
| K7ZJP7 | IgM heavy chain constant region (Fragment) | 0 | 1.28 |
| P27594 | Interferon-induced GTP-binding protein Mx1 | 1.35E-30 | 1.22 |
| Q29550 | Liver carboxylesterase | 1.30E-22 | 1.97 |
| Q9N1X3 | Lung surfactant protein A (Fragment) | 2.03E-69 | 1.49 |
| P12069 | Lysozyme C-3 | 1.12E-26 | 1.32 |
| P79379 | Metallothionein-2A | 4.39E-08 | 1.75 |
| A0A0B8RSL6 | Methyltransferase like 7A | 2.66E-11 | 1.24 |
| K9LR63 | MHC class I antigen (Fragment) | 0.027493846 | 1.45 |
| A0A0A7BZS1 | MHC class I antigen | 1.01E-09 | 2.45 |
| Q8HX70 | MHC class II antigen (Fragment) | 2.46E-17 | 1.96 |
| A7ISM6 | MHC class II antigen alpha chain | 1.54E-16 | 1.28 |
| A7ISN2 | MHC class II antigen alpha chain | 6.50E-15 | 2.76 |
| Q4ZJG0 | MHC class II antigen | 5.63E-05 | 1.25 |
| G0KXR0 | MHC class II antigen | 7.47E-42 | 1.34 |
| Q31081 | MHC class II SLA-DRB1-4 (Fragment) | 2.74E-07 | 1.75 |
| F1S9I9 | MOSC domain-containing protein 2, mitochondrial | 3.98E-09 | 1.44 |
| A0A0B8RSG9 | Oxysterol-binding protein | 0.007599567 | 1.46 |
| C3W8F2 | Perilipin | 0.003938598 | 1.23 |
| F1SID1 | Promyelocytic leukemia | 4.36E-12 | 1.22 |
| Q863Z0 | Proteasome activator complex subunit 2 | 1.49E-93 | 1.40 |
| Q9N1X4 | Pulmonary surfactant-associated protein D | 3.24E-26 | 1.30 |
| I3L6D2 | Ras-related protein Rab-1A (Fragment) | 1.69E-07 | 1.26 |
| Q06AU0 | RBM4 | 0.003890524 | 1.23 |
| Q1L128 | Secretoglobin family 1A member 1 (Fragment) | 3.01E-22 | 2.29 |
| Q9TQT5 | SLA-DR1 beta1 domain (Fragment) | 0.000177218 | 1.57 |
| Q1XFL4 | Surfactant protein C | 2.11E-15 | 1.26 |
| J9JIM9 | Testin (Fragment) | 0.000105822 | 1.21 |
| F1S8G5 | Thimet oligopeptidase | 4.04E-14 | 1.28 |
| F1SR25 | Thromboxane-A synthase | 0.006358419 | 1.24 |
| I3LPN0 | Tryptase | 1.41E-20 | 1.83 |
| F4ZS20 | Ubiquitin-conjugating enzyme | 2.46E-05 | 1.25 |

*Fold change is relative to LPS+HA (sham)-treated pigs, so a fold change≥1.2 represents more protein abundance in LPS+HA-treatment pigs.
